# NAT10 promotes the progression of clear cell renal cell carcinoma by regulating ac4C acetylation of NFE2L3 and activating AKT/GSK3β signaling pathway

**DOI:** 10.1038/s41419-025-07528-w

**Published:** 2025-04-02

**Authors:** Zenghui Sun, Yuqiong Wang, Chao Zheng, Lixiang Xiao, Yuanwei Zang, Liang Fang, Xixi Cui, Mingjie Chang, Qiyu Sun, Wenjuan Li, Juchao Ren

**Affiliations:** 1 Key Laboratory for Experimental Teratology of Chinese Ministry of Education, The Shandong Provincial Key Laboratory of Infection and Immunology, Department of Pathogenic biology, School of basic medical sciences, Shandong University, Jinan, PR China; 2 Department of Urology, Qilu Hospital, Shandong University, Jinan, PR China

**Keywords:** Renal cell carcinoma, Cell biology

## Abstract

Clear cell renal cell carcinoma (ccRCC) is the most common histological subtype of renal cell carcinoma, and the tumour growth and metastasis of ccRCC are related to prognosis. N4-acetylcytidine (ac4C) is one of the major modifications of RNA and is known to be mediated by N-acetyltransferase 10 (NAT10). The role of NAT10 in cancer is gradually being revealed, although the role of NAT10-mediated RNA ac4C modification in ccRCC has not been reported. In this study, NAT10 was found to be upregulated in ccRCC tissues and associated with a poor prognosis in patients. HIF-1α activated NAT10 expression at the transcriptional level. CCK8, EdU, Transwell and scratch assays after NAT10 knockdown or overexpression showed that NAT10 promoted cell proliferation and migration. The results of subcutaneous xenograft and caudal vein injection showed that NAT10 promoted tumour growth and metastasis in vivo, while Remodelin inhibited tumour growth. The acRIP-seq, RIP, RNA stability and dual luciferase reporter experiments showed that NAT10 activated ac4C acetylation of NFE2L3 mRNA and promoted NFE2L3 mRNA stability. The ChIP-seq results showed that NFE2L3 regulated the expression of LASP1 and thus activated the AKT/GSK3β signalling pathway. In summary, our results suggest that NAT10 mediates ac4C acetylation of NFE2L3 mRNA, promotes its mRNA stability, regulates the LASP1-AKT/GSK3β/β-catenin axis and promotes the progression of renal clear cell carcinoma.

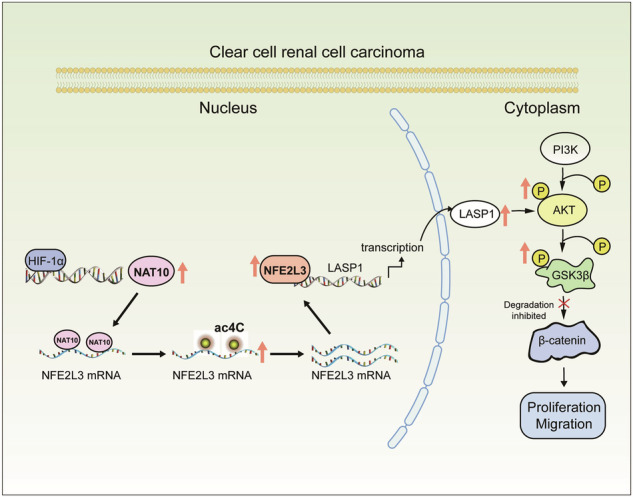

## Introduction

Renal cell carcinoma (RCC) is one of the most common urogenital cancers. In 2020, there were more than 400,000 new diagnoses of kidney cancer, causing nearly 180,000 deaths worldwide [1]. Clear cell renal cell carcinoma (ccRCC) is the most common histological subtype, accounting for 75–80% of all renal cell carcinomas [2], and has a lower survival rate than papillary renal cell carcinoma and renal chromophobe cell carcinoma [3]. Many patients with RCC are first diagnosed at an advanced stage, and the overall prognosis is poor. Tumour metastasis is the most dangerous factor and determines the treatment and prognosis of RCC patients. Systemic therapy for metastatic clear cell renal cell carcinoma includes targeted therapy and immunotherapy. However, the 5-year survival rate of metastatic renal cell carcinoma is still approximately 10% [4]. Therefore, it is of great significance to find effective molecular diagnostic markers and therapeutic targets for the intervention of ccRCC metastasis and progression [5].

Posttranscriptional mRNA carries a variety of chemical modifications that play an important role in gene expression. The known internal mRNA modifications include base methylation, such as N6-methyladenosine (m6A), N1-methyladenosine (m1A), 5-methylcytosine (m5C), 5-hydroxymethylcytosine (hm5C), 2-O methylation (Nm), and uridine to pseuduridine isomerization (ψ). Abnormal regulation of RNA epigenetics is a key factor affecting tumour progression [6–8]. Arango and colleagues found that N4-cytidine acetylation (ac4C) is a novel mRNA modification that can improve the stability and may promote translation efficiency of transcripts [9]. Ac4C was initially found to exist in the first anticodon of bacterial tRNAmet [10] and was essential for coding accuracy in protein synthesis [11]. In eukaryotic RNA, ac4C was found to exist at specific sites of tryptophan and leucine tRNAs and 18S rRNA [12, 13] and was catalyzed by NAT10 and its homologous proteins, which were identified as the acetyltransferases of ac4C in human tRNA and rRNA [14]. In 2016, researchers used mass spectrometry to show that ac4C also existed in human mRNA [15], and ac4C acetylation has been shown to promote the stability of mRNA, influence tRNA selection and thus regulate mRNA translation [9]. According to recent reports, the high expression of NAT10 indicates a poor prognosis of malignant tumours. NAT10-mediated ac4C of COL5A1 promotes metastasis and EMT in gastric cancer [16], and NAT10-mediated ac4C acetylation of mRNA promotes the development of bladder cancer [7]. Although NAT10-mediated mRNA ac4C modification has been continuously studied, the role of mRNA ac4C acetylation in ccRCC remains unknown.

Our study found that NAT10 was highly expressed in ccRCC and correlated with a poor prognosis in ccRCC patients. NAT10 mediated ac4C modification of NFE2L3 to promote its mRNA stability, thus promoting the proliferation and migration of ccRCC cells by regulating the LASP1/AKT signalling pathway. Our study revealed that the NAT10-NFE2L3-LASP1-AKT/GSK3β axis plays a carcinogenic role in the progression of ccRCC.

## Results

### Increased NAT10 expression is associated with a poor prognosis in patients with ccRCC

**Fig. 1:**
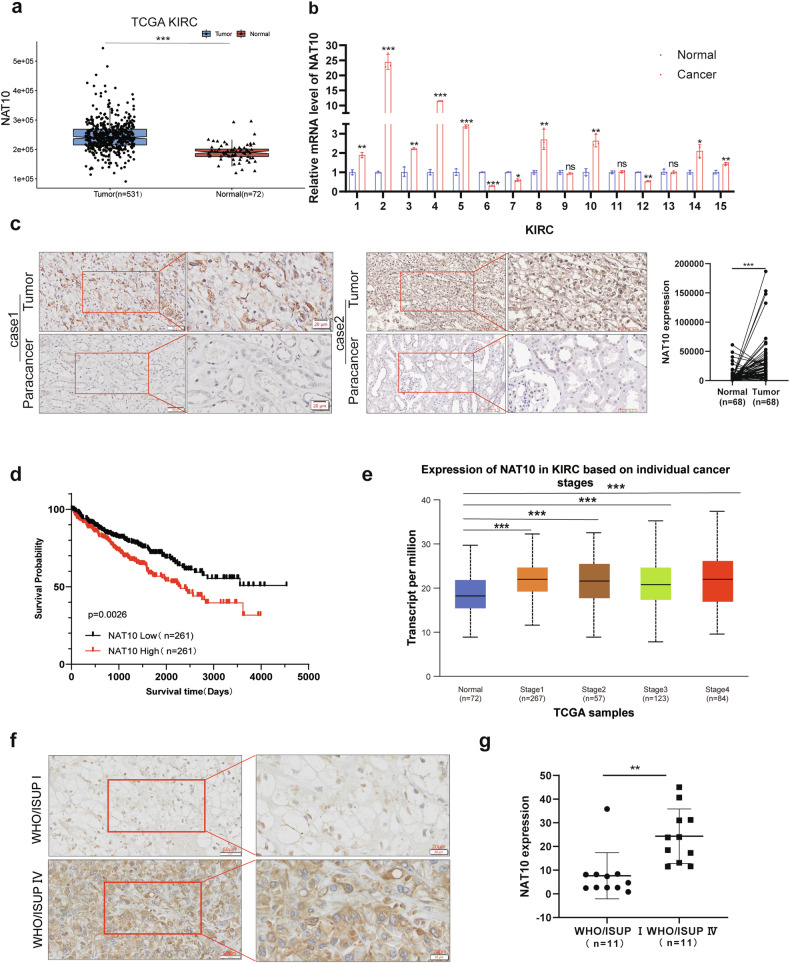
Increased NAT10 expression is associated with a poor prognosis in patients with ccRCC. **a** The TCGA database of Sangerbox 3.0 was used to analyze the expression levels of NAT10 in KIRC cancer tissues (*n* = 531) and normal tissues (*n* = 72). **b** QRT-PCR was used to detect NAT10 mRNA levels in 15 pairs of ccRCC cancer tissues and adjacent para-cancer tissues. **c** Left: Representative images of in situ NAT10 expression levels in 68 pairs of ccRCC cancer tissues and adjacent tissues detected by immunohistochemical staining. Scale: 50 μm (left); 20 μm (right). Statistical analysis of the intensity of immunohistochemical staining (right). **d** Kaplan–Meier analysis survival for ccRCC patients based on NAT10 expression (*n* = 261, *p* = 0.0026, log-rank test). **e** UALCAN was used to analyze the expression level of NAT10 in normal tissues and in each stage of ccRCC. **f** Comparison of expression levels of NAT10 in ccRCC WHO/ISUP stage I and IV by immunohistochemical staining. WHO/ISUP stage I(above), WHO/ISUP stage IV(below). Scale: 50 μm(left); 20 μm(right). **g** Statistical analysis of the intensity of immunohistochemical staining.

TCGA KIRC data showed that compared with normal tissues, the expression of NAT10 in ccRCC was significantly increased (*p* < 0.05) (Fig. 1a, Supplementary Fig. S1g). By extracting RNA from ccRCC tissues and corresponding adjacent tissues, we found that NAT10 was highly expressed in most ccRCC tissues, which was consistent with the results of the database (Fig. 1b). Immunohistochemical (IHC) staining results were consistent with the above results, showing that the expression of NAT10 in ccRCC tissues was higher than that in paracancerous tissues (Fig. 1c). Kaplan‒Meier analysis showed that higher NAT10 expression was associated with a poor prognosis in ccRCC patients (Fig. 1d). In addition, UALCAN (http://ualcan.path.uab.edu/analysis.html) and TISID8 data (http://cis.hku.hk/TISIDB/) showed that the expression of NAT10 in ccRCC was significantly higher in WHO/ISUP stage IV than in WHO/ISUP stage I (Fig. 1e, Supplementary Fig. S1h), and the immunohistochemical results were also consistent with the above results (Fig. 1f, g). Analysis of the IHC staining results showed that NAT10 expression levels were indeed correlated with WHO/ISUP staging of ccRCC (Supplemental Table 1). In summary, these results suggest that the increased expression of NAT10 in ccRCC is associated with the progression of ccRCC and a poor prognosis in patients.

### HIF-1α regulates and promotes NAT10 transcription in ccRCC

**Fig. 2:**
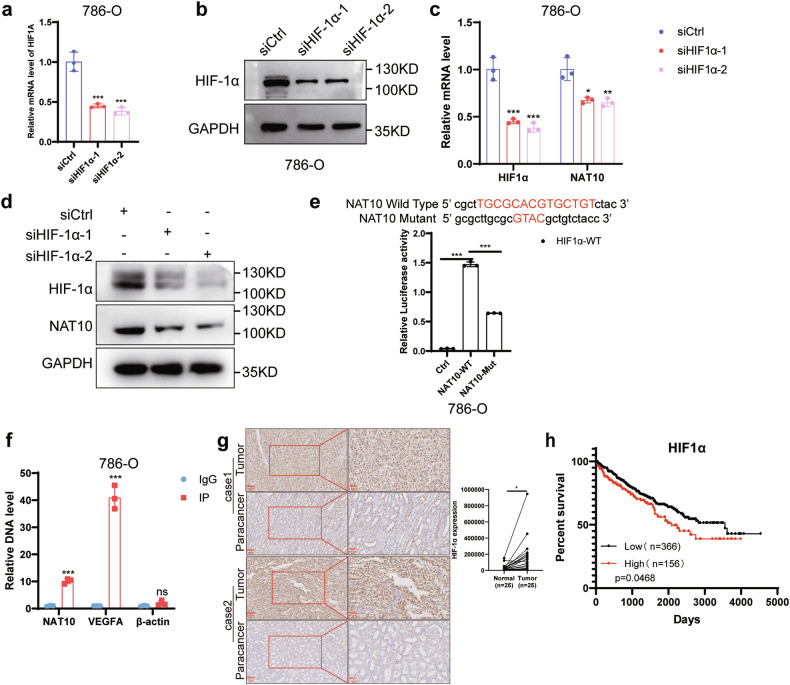
HIF-1α regulates and promotes NAT10 transcription in ccRCC. **a** The effect of HIF-1α-specific small interfering RNA on mRNA knockdown in 786-O cells by qRT-PCR. **b** Western blot analysis of HIF-1α protein knockdown in 786-O cells.**c** The level of NAT10 mRNA in 786-O cells was also decreased after HIF-1α knockdown by qRT-PCR. **d** Western blot analysis showed that the level of NAT10 protein decreased after HIF-1α knockdown in 786-O cells. **e** Dual luciferase reported that HIF-1α was overexpressed, the relative activity of firefly luciferase fused with the wild-type or NAT10 promoter (0, +300) mutant plasmid in 786-O cells. **f** ChIP-qPCR showed that HIF-1α can bind to the NAT10 promoter region (0, +300), VEGFA was the positive control and β-actin was the negative control. **g** Left: Representative images of in situ HIF-1α expression levels in 25 pairs of ccRCC cancer tissues and adjacent tissues detected by immunohistochemical staining. Scale: 100μm (left); 50 μm (right). Statistical analysis of the intensity of immunohistochemical staining (right). **h** Kaplan–Meier analysis survival for ccRCC patients based on HIF-1α expression (*p* = 0.0468, log-rank test).

To explore the regulatory mechanism of increased NAT10 expression in ccRCC, the PROMO website (http://alggen.lsi.upc.es/cgi-bin/promo_v3/promo/promoinit.cgi?dirDB=TF_8.3) was used to predict the transcription factors that could bind to the NAT10 promoter. The genetic factor leading to ccRCC is primarily mutation of the VHL gene, resulting in the accumulation of the HIF-1α and HIF-2α transcription factors [17, 18]. The PROMO website predicted that there were potential HIF-1α binding sites in the promoter region of NAT10. Therefore, we explored whether HIF-1α regulates the increased expression of NAT10 in ccRCC. We used HIF-1α-specific small interfering RNA to knockdown HIF-1α mRNA and protein expression (Fig. 2a, b; Supplementary Fig. S1j) and found that HIF-1α knockdown resulted in decreased NAT10 expression levels (Fig. 2c, d). Chromatin immunoprecipitation (ChIP) assays revealed the enrichment of HIF-1α transcription factors in the NAT10 promoter region, with VEGFA as a positive control and β-actin as a negative control (Fig. 2f). In addition, the results of the dual luciferase assay showed that the luciferase activity of the NAT10 promoter wild-type plasmid was significantly higher than that of the NAT10 promoter mutant plasmid (Fig. 2e). Hypoxia significantly increased NAT10 promoter luciferase activity, and NAT10 promoter activity decreased following HIF-1α knockdown under normal and hypoxia conditions, but the mutant promoter luciferase activity was not affected (Supplementary Fig. S1i). VEGFA and LDHA are the known downstream genes of HIF-1α. We found that the expression of NAT10 in ccRCC tissues was positively correlated with the expression of VEGFA and LDHA (Supplementary Fig. S1k). NAT10 expression was positively correlated with HIF-1α expression in ccRCC by analyzing the IHC results and using GEPIA website (Supplementary Fig. S1l, m). IHC results showed that HIF-1α expression was significantly increased in ccRCC (Fig. 2g). High expression of HIF-1α was associated with a poor prognosis in patients with ccRCC (Fig. 2h). The above data indicate that HIF-1α promotes the transcription of NAT10 in ccRCC, which may be one of the reasons for the high expression of NAT10.

### NAT10 promotes the proliferation and migration of ccRCC

**Fig. 3:**
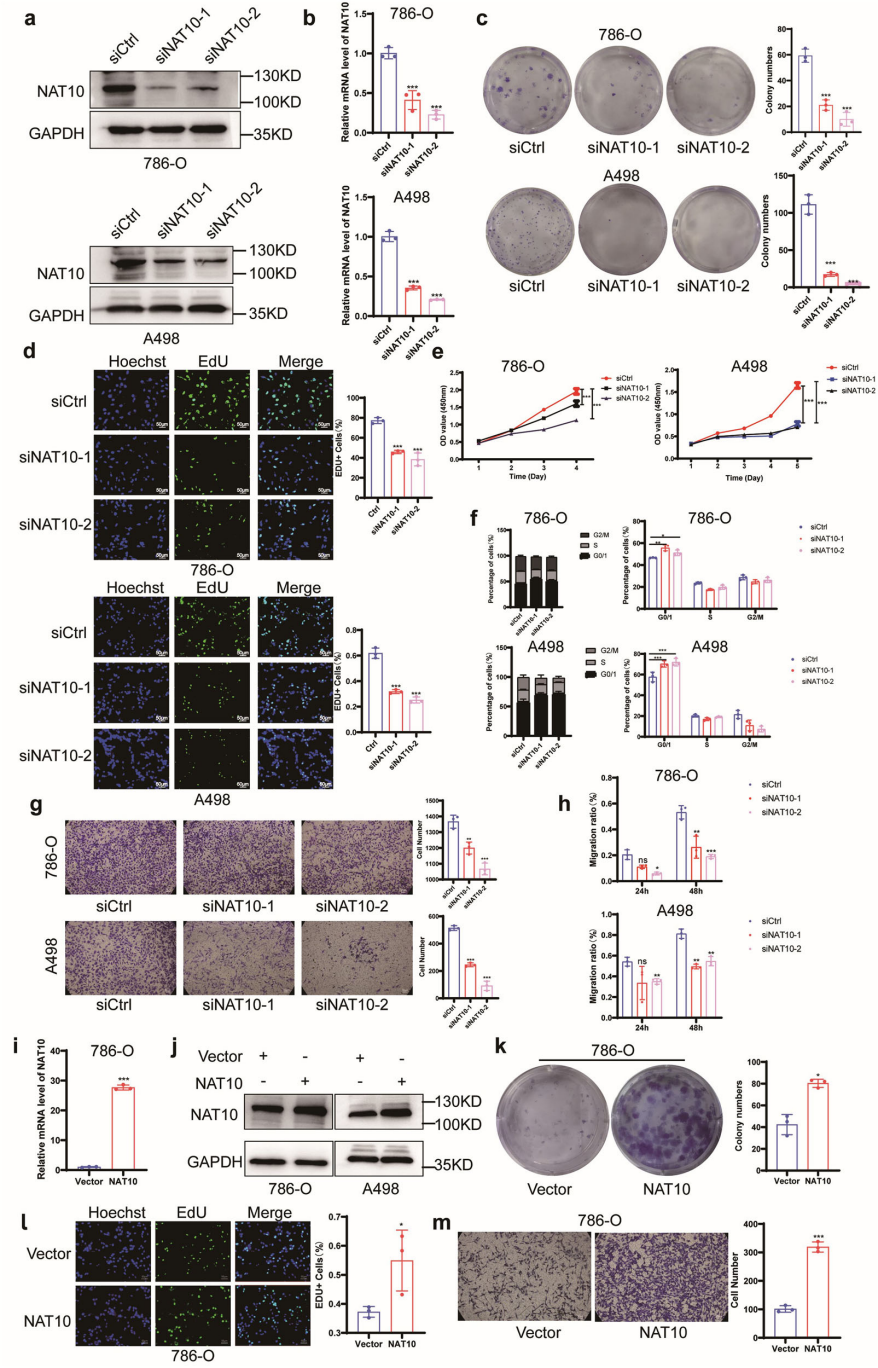
NAT10 promotes proliferation and migration of ccRCC in vitro. **a** Western blot was used to detect the knockdown effect of NAT10 interfering protein in 786-O and A498 cells. **b** The knockdown effect of NAT10 small interference mRNA in 786-O and A498 cells by qRT-PCR. **c** Left: Colony formation assayed evaluating colony generating ability. Right: quantitative analysis of the capacity of cell colony generation. **d** Left: EdU assayed evaluating cell proliferation. Right: Quantified analysis of EdU^+^ proliferating cells. Scale: 50 μm. **e** CCK8 assayed evaluating cell proliferation. **f** Flow cytometry showed that G0/G1 phase cycle arrest occurred after NAT10 knockdown. **g** Left: Transwell assayed evaluating cell migration. Right: quantitative analysis of migrating cells. Scale: 50 μm. **h** The migration ability of 786-O and A498 cells was significantly inhibited at 12 h and 24 h after NAT10 knockdown by scratch assay. **i** The overexpression of NAT10 mRNA in 786-O cells by qRT-PCR. **j** The levels of NAT10 overexpressed protein by western blot. **k** Overexpression of NAT10 promotes the colony generation of 786-O cells. **l** Left: EdU assayed evaluating cell proliferation by NAT10 overexpression. Right: Quantification of cell proliferation. Scale: 50 μm. **m** Left: Transwell evaluated cell migration by NAT10 overexpression. Right: quantitative analysis of migrating cells. Scale: 50 μm.

To explore the role of NAT10 in ccRCC, we constructed ccRCC cell lines (786-O and A498) with NAT10 knockdown (Fig. 3a, b). Downregulation of NAT10 significantly inhibited colony formation (Fig. 3c) and proliferation of 786-O cells and A498 cells (Fig. 3d, e). To confirm whether NAT10 can regulate the cell cycle, we used flow cytometry, which showed that NAT10 knockdown increased the percentage of G0/G1 phase in 786-O cells and A498 cells; that is, downregulation of NAT10 can cause G0/G1 cell cycle arrest (Fig. 3f; Supplementary Fig. S1a). When NAT10 was knocked down in 786-O cells and A498 cells, their migration ability was significantly inhibited (Fig. 3g, h; Supplementary Fig. S1b). To clarify the role of NAT10 in ccRCC, NAT10 wild-type plasmids were transfected into 786-O cells and A498 cells to construct NAT10 overexpression cell lines (Fig. 3i, j; Supplementary Fig. S1c). The overexpression of NAT10 significantly promoted the colony formation and proliferation of 786-O cells and A498 cells (Fig. 3k, l; Supplementary Fig. S1d, e), which also promoted the ability of cells to migrate (Fig. 3m; Supplementary Fig. S1f). These results suggest that NAT10 may play a role in promoting cancer in ccRCC, promoting the proliferation and migration of ccRCC cells.

### NAT10 promotes tumour growth and metastasis in vivo

**Fig. 4:**
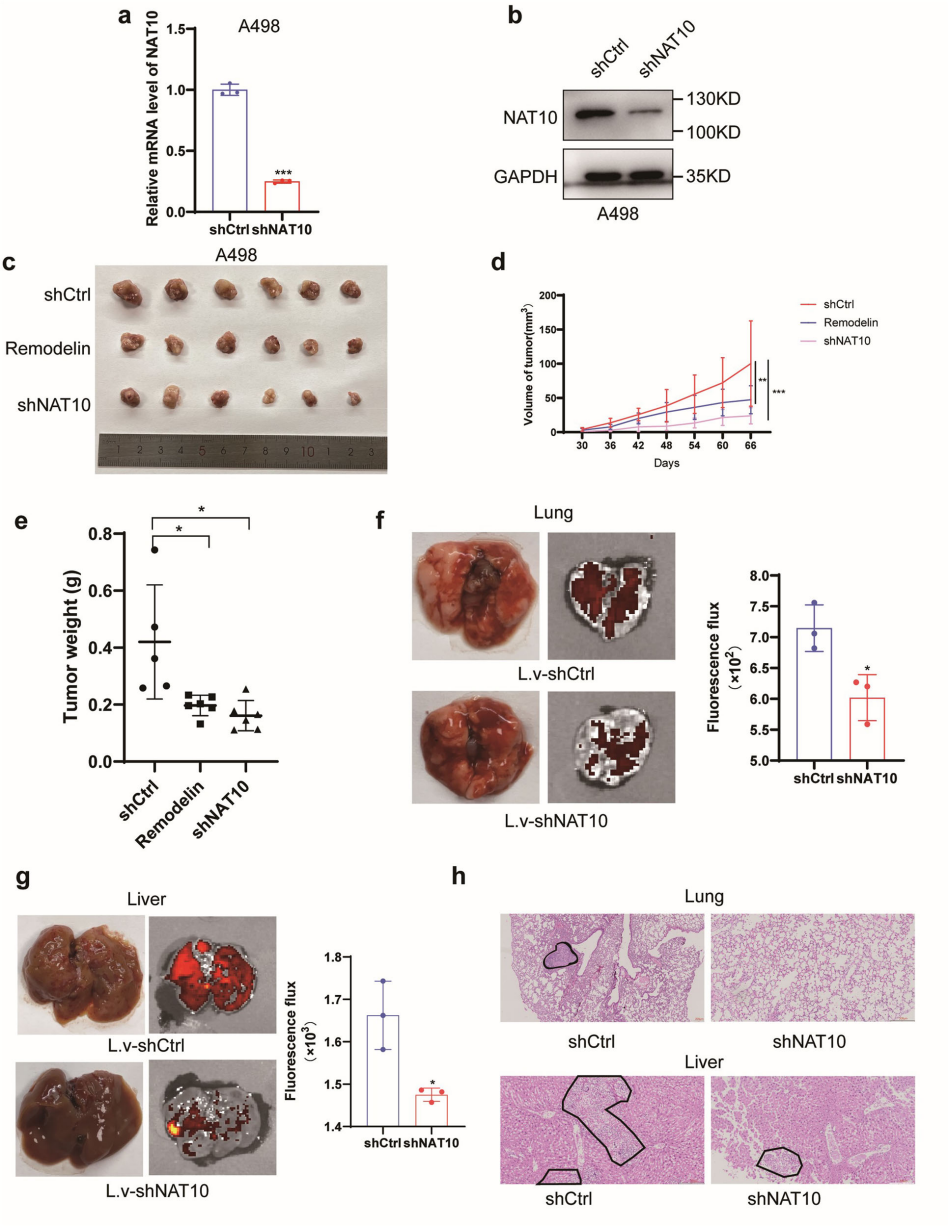
NAT10 promotes tumour growth and metastasis in vivo. **a** The effect of NAT10 knockdown detected by qRT-PCR in A498 cells. **b** The protein level of NAT10 knockdown detected by western blot. **c** Representative images of subcutaneous tumours formed by the indicated cells. **d** The tumour volume of nude mice was monitored every 6 days. **e** Quantitative analysis of subcutaneous tumour after 66 days. **f** Left: Representative image of metastatic lung tumour. Right: quantitative analysis of fluorescence intensity of lung metastasis. **g** Left: Representative image of metastatic liver tumour. Right: quantitative analysis of fluorescence intensity of liver metastasis. **h** HE staining results of metastatic lung tumours (above) and metastatic liver tumours (below).

To explore the role of NAT10 in vivo, the constructed A498 cells with stable NAT10 knockdown (Fig. 4a, b) were used for tumour xenotransplantation. Compared with the control group, the NAT10 knockdown group showed a significant inhibition in the growth of tumours, and the group treated with the NAT10 inhibitor Remodelin also showed a significant inhibition in the growth of tumours (Fig. 4c–e). Dot Blot experiment showed that Remodelin inhibited RNA ac4C modification in ccRCC cells (Supplementary Fig. S1n). To further investigate the effect of NAT10 on tumour metastasis in vivo, A498 cells with stable NAT10 knockdown and control cells were injected into nude mice through the caudal vein. Forty days later, liver and lung imaging results showed that downregulation of NAT10 inhibited ccRCC metastasis (Fig. 4f, g). HE staining showed that the number of liver and lung metastases after NAT10 knockdown was significantly less than that in the control group, which was consistent with previous results (Fig. 4h). In conclusion, upregulation of NAT10 plays an important role in promoting the proliferation and metastasis of ccRCC.

### NAT10 interacts with NFE2L3 and promotes its stability

**Fig. 5:**
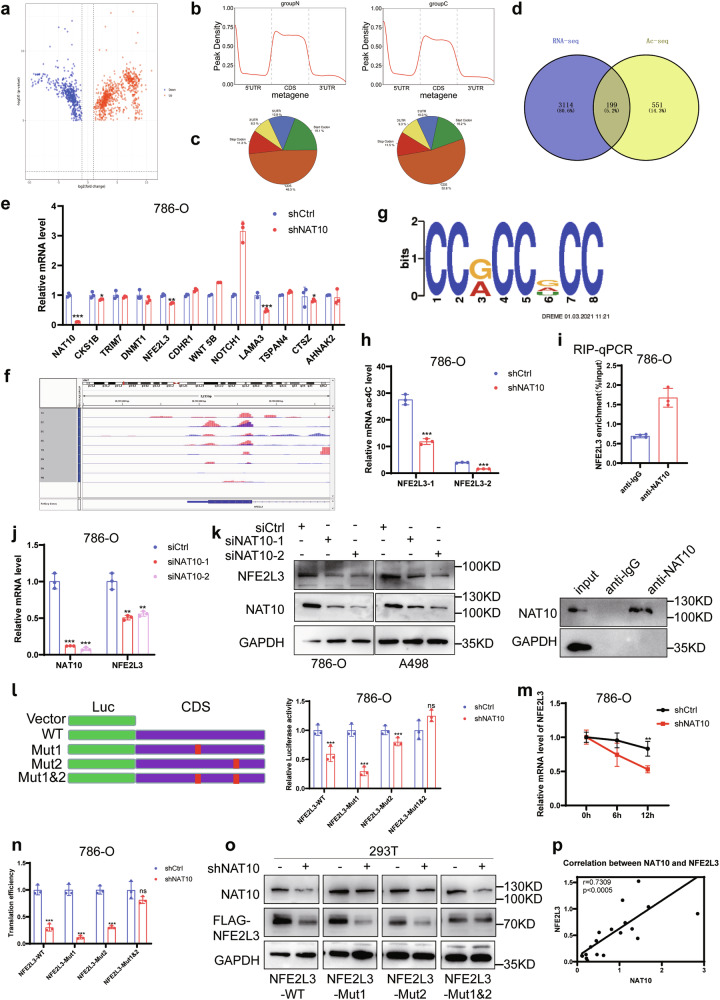
NAT10 interacts with NFE2L3 and promotes its stability. **a** The acetylation levels of different genes with *p* < 0.05 were analyzed to make a volcano map. Blue: decrease in ac4C acetylation level; red: increase in ac4C acetylation level (*n* = 4). **b**, **c** ac4C acetylation of genes mainly occurs in the CDS region. **d** There were 199 gene intersections between RNA-seq and acRIP-seq of genes with increased expression in ccRCC and genes with increased ac4C acetylation in cancer. **e** mRNA variation trend of 11 candidate genes after NAT10 knockdown in 786-O cells. **f** The abundance of ac4C on NFE2L3 mRNA transcripts in ccRCC cancer tissues was detected by acRIP-seq. **g** Overall analysis of ac4C in ccRCC tissues Main common sequence motifs identified in ac4C peaks. **h** acRIP-qPCR revealed ac4C acetylation on NFE2L3 mRNA. **i** RIP-qPCR showed that NAT10 could bind to NFE2L3 mRNA. Below is an anti-NAT10 that specifically pulls down the NAT10 protein. **j**–**k** QRT-PCR and western blot showed that NFE2L3 mRNA and protein level decreased after NAT10 knockdown. **l** The activity of NFE2L3-wild type and mutant vectors after NAT10 knockdown were detected by dual luciferase. **m** RNA attenuation experiment showed that the stability of NFE2L3 mRNA decreased after NAT10 knockdown. **n** The role of NAT10 on NFE2L3 mRNA translation efficiency. (evaluated using relative fluorescence intensity/relative RNA expression [16]). **o** Western blot detection of Flag levels in control group and knockdown NAT10 group verified the effect of NAT10-mediated ac4C modification on NFE2L3 protein. **p** The correlation of NAT10 and NFE2L3 mRNA in 20 ccRCC tissues was performed, *r* = 0.7309, *p* < 0.005.

To determine the role of NAT10 in promoting the progression of ccRCC, we performed RNA sequencing (RNA-Seq) and ac4C-modified RNA immunoprecipitation sequencing (acRIP-seq) on four pairs of ccRCC tissues and their adjacent tissues. The volcano map and heat map showed both increased and decreased mRNA acetylation (Fig. 5a; Supplementary Fig. S2a), and ac4C modification sites mainly existed in the CDS region of transcripts (Fig. 5b, c). A comprehensive analysis of the acRIP-seq and RNA-seq showed that the expression and acetylation levels of 199 transcripts were increased in ccRCC tissues (Fig. 5d). Since ac4C modification can improve mRNA stability, the 11 candidate genes which had higher level of ac4C modification and mRNA expression in ccRCC tissues were examined, and the results showed that only the NFE2L3 mRNA levels decreased in 786-O cells and A498 cells after NAT10 knockdown (Fig. 5e; Supplementary Fig. S2b). Therefore, we decided to study the mechanism of action of NFE2L3 in ccRCC. The acRIP-seq results showed that the abundance of ac4C in NFE2L3 was significantly upregulated in cancer tissues (Fig. 5f), that ac4C modification mainly occurred in the common sequences of CCHCCRCC (H = G or A, R = G or A or U) (Fig. 5g), and that the ac4C modification sites all existed in the CDS region of NFE2L3. acRIP-qPCR was used to verify the results of the acRIP-seq. After NAT10 knockdown, NFE2L3 mRNA enriched with an ac4C-specific antibody was significantly reduced (Fig. 5h). RIP-qPCR assays showed that, compared with the control antibody IgG, the NAT10-specific antibody could significantly enrich NFE2L3 mRNA (Fig. 5i), indicating that NAT10 could directly specifically bind NFE2L3 mRNA and induce ac4C modification. We wanted to determine whether NAT10 can regulate NFE2L3, and the results showed that after NAT10 knockdown, the NFE2L3 mRNA levels (Fig. 5j; Supplementary Fig. S2c) and protein levels were reduced (Fig. 5k). To determine whether NAT10 regulates the expression of NFE2L3 mRNA by acetylation modification, NFE2L3 wild-type, acetylation site 1 mutant, acetylation site 2 mutant and acetylation site 1&2 mutant (the common acetylation sequence C mutated to G) plasmids were constructed. Dual luciferase reporter assay results showed that NAT10 knockdown reduced the luciferase activity of the wild-type NFE2L3, site 1 mutant and site 2 mutant but had no effect on the site 1&2 mutant (Fig. 5l; Supplementary Fig. S2d). To further clarify the specific regulatory role of NAT10-mediated acetylation on NFE2L3 mRNA, the half-life and translation efficiency (evaluated using relative fluorescence intensity/relative RNA expression [16]) of NFE2L3 mRNA were determined after NAT10 knockdown. The results showed that the stability of NFE2L3 mRNA was reduced after NAT10 knockdown (Fig. 5m; Supplementary Fig. S2e), which also reduced its translation efficiency (Fig. 5n; Supplementary Fig. S2f). To further determine whether NAT10 regulates its expression through acetylation of NFE2L3 mRNA, Flag-tagged NFE2L3 wild-type, site 1 mutant, site 2 mutant and site 1&2 mutant plasmids were constructed. When NAT10 was knocked down, expression of Flag-tagged wild-type NFE2L3, site 1 mutant and site 2 mutant in 293 T and A498 cells was significantly decreased, but no effect was found on the site 1&2 mutant (Fig. 5o; Supplementary Fig. S2g). The expression of NFE2L3 in ccRCC tissues is positively correlated with the expression of NAT10 (Fig. 5p). In addition, NAT10 does not bind to NFE2L3 protein (Supplementary Fig. S2h). We used NFE2L3 RNA probes to detect the presence of co-localization of NFE2L3 RNA and NAT10 protein in ccRCC tissues and 786-O cells (Supplementary Fig. S2i, j). In summary, our results suggest that NAT10 mediates ac4C modification of NFE2L3 mRNA to promote its stability and may also promote its translational efficiency.

### NAT10 promotes the progression of ccRCC by regulating NFE2L3

**Fig. 6:**
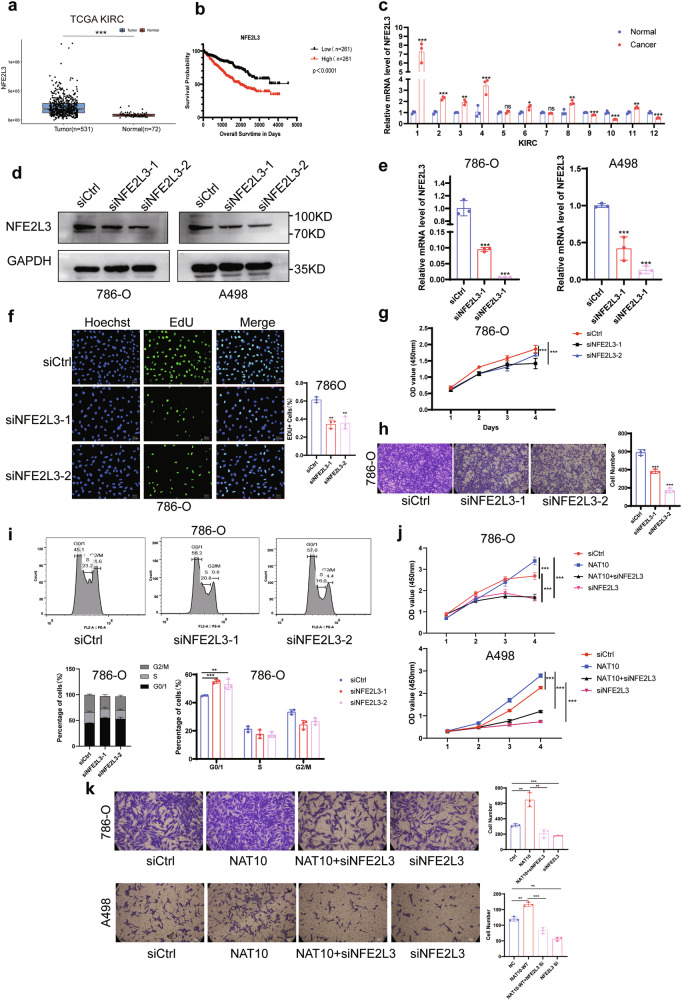
NAT10 promotes the progression of ccRCC by regulating NFE2L3. **a** Relative expression of NAT10 in KIRC and normal tissues in GEO datasets. **b** Kaplan-Meier survival analysis of NFE2L3 expression in ccRCC patients (*n* = 261, *p* < 0.0001, log-rank test). **c** QRT-PCR was used to detect NFE2L3 mRNA levels in 12 pairs of ccRCC cancer tissues and adjacent tissues. **d** Western blot analysis of NFE2L3 knockdown efficiency in 786-O and A498 cells. **e** The knockdown effect of NFE2L3 small interference RNA in 786-O and A498 cells by qRT-PCR. **f** Left: EdU assayed evaluating cell proliferation. Right: Quantitative analysis of EdU^+^ proliferating cells. Scale: 50 μm. **g** CCK8 assayed evaluating cell proliferation. **h** Left: Transwell assayed evaluating cell migration. Right: Quantitative analysis of migrating cells. Scale: 50 μm. **i** Flow cytometry showed that G0/G1 phase cycle arrest occurred after NFE2L3 knockdown. **j** The proliferation ability of 786-O and A498 cells after overexpression of NAT10 and transfection of NFE2L3 siRNAs was detected by CCK8 assays. **k** Left: The migration ability of 786-O and A498 cells after overexpression of NAT10 and transfection of NFE2L3 siRNAs was detected by Transwell assays. Right:quantitative analysis of migrating cells. Scale: 50μm.

NAT10 expression were positively correlated with NFE2L3 expression in ccRCC by analyzing the IHC results and using GEPIA website (Supplementary Fig. S2l, m). The TCGA KIRC database and IHC results showed that NFE2L3 was highly expressed in ccRCC tissues (Fig. 6a; Supplementary Fig. S2k), and its high expression was associated with a poor prognosis in ccRCC patients (Fig. 6b). To verify the above results, we examined the expression of NFE2L3 in cancer tissues of 12 pairs of ccRCC patients and found by qRT‒PCR that it was generally higher than that in the adjacent tissues (Fig. 6c). To further determine the role of NFE2L3 in ccRCC, NFE2L3 knockdown cell lines were constructed (Fig. 6d, e), and we found that NFE2L3 knockdown significantly inhibited cell proliferation (Fig. 6f, g; Supplementary Fig. S3a, b) and migration (Fig. 6h; Supplementary Fig. S3c). To determine whether NFE2L3 can also regulate the cell cycle, flow cytometry was used, and the results showed that NFE2L3 knockdown increased the percentage of G0/G1 phase in 786-O cells and A498 cells (Fig. 6i; Supplementary Fig. S3d); that is, downregulation of NFE2L3 can cause G0/G1 cell cycle arrest, which is consistent with the role of NAT10 in ccRCC. In addition, downregulation of NFE2L3 expression significantly inhibited the proliferation and migration of ccRCC cells induced by NAT10 overexpression (Fig. 6j, k). Therefore, our experimental data suggest that NAT10 promotes the malignant progression of ccRCC by upregulating the expression of NFE2L3.

### NFE2L3 activates the PI3K/AKT pathway by regulating LASP1

**Fig. 7:**
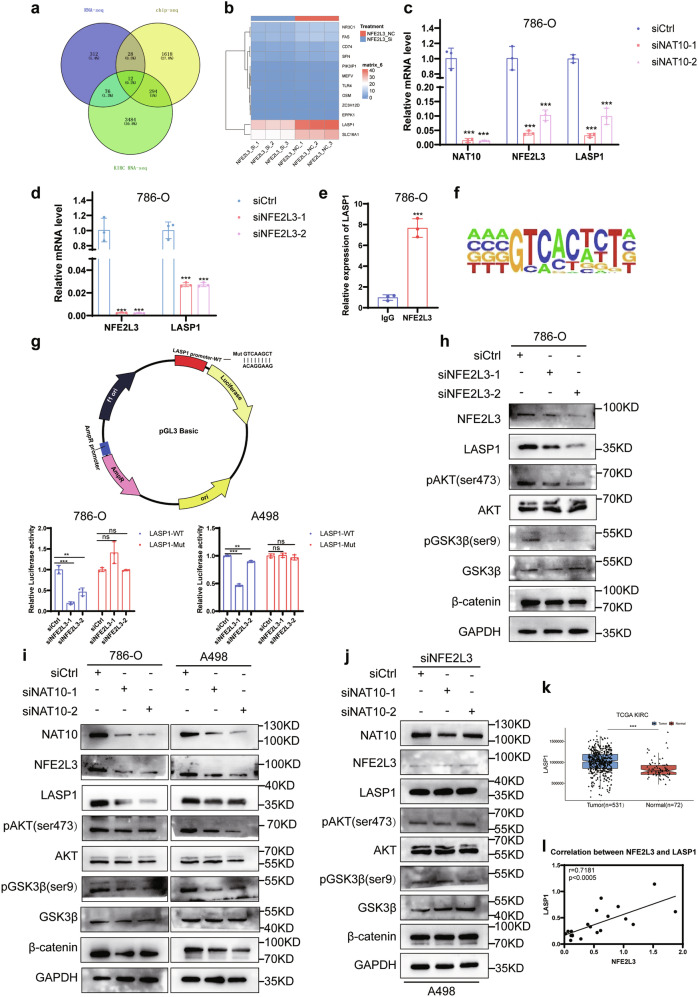
NFE2L3 activates the PI3K/AKT pathway by regulating LASP1. **a** Compared with the control group, RNA-seq results showed that 312 transcripts in the NFE2L3 knockdown group were downregulated and 3 114 transcripts in the ccRCC tissue were upregulated, and ChIP-seq showed that NFE2L3 could bind to the promoter region of 1 618 transcripts. There were 12 overlapping transcripts. **b** The expression levels of 12 overlapping transcripts in control cells and knockdown NFE2L3 cells were analyzed by heat map. **c** The mRNA of NFE2L3 and LASP1 decreased after NAT10 knockdown in 786-O cells by QRT-PCR. **d** QRT-PCR detected the decrease of LASP1 mRNA after NFE2L3 knockdown in 786-O cells. **e** NFE2L3 was detected by ChIP-qPCR to bind to the promoter of LASP1. **f** The binding sites of NFE2L3 and LASP1 transcripts mainly occur in the common sequences. **g** Dual luciferase assay showed that NFE2L3 could regulate the LASP1 promoter region. **h** After NFE2L3 knockdown, LASP1 and β-catenin expression was decreased, AKT/GSK3β phosphorylation was inhibited. **i** After NAT10 knockdown, the expressions of NFE2L3, LASP1 and β-catenin were decreased, the phosphorylation of AKT/GSK3β was inhibited. **j** The expression of LASP1 and phosphorylation of AKT/GSK3β were not changed after NAT10 knockdown in the NFE2L3 knockdown cells, nor was the expression of β-catenin. **k** The TCGA database showed that LASP1 was highly expressed in ccRCC. **l** The correlation of NFE2L3 and LASP1 mRNA in 20 ccRCC tissues was performed, *r* = 0.71181, *p* < 0.0005.

We wanted to determine how NAT10 regulates NFE2L3 to promote the progression of ccRCC, and since NFE2L3 is a transcription factor [19], we performed ChIP-seq for NFE2L3, as well as RNA-seq in 786-O cells with a NFE2L3 knockdown and control 786-O cells. The results of the RNA-seq showed that 312 transcripts in the NFE2L3 knockdown group were downregulated, and 3 114 transcripts in the ccRCC tissue described above were upregulated. Moreover, after comprehensive analysis of the ChIP-seq results, there were 12 overlapping transcripts (Fig. 7a). Analysis of the expression levels of the 12 transcripts in control cells and NFE2L3 knockdown cells (Fig. 7b) showed that the expression level of LASP1 was the highest in cells, and therefore we hypothesized that NFE2L3 promoted the progression of ccRCC by affecting LASP1 (Supplementary Fig. S4a). We wanted to determine whether NAT10 and NFE2L3 can regulate LASP1, and our results showed that knocking down both NAT10 and NFE2L3 could reduce the mRNA level of LASP1 (Fig. 7c, d; Supplementary Fig. S4b, c) and the protein levels (Fig. 7h, i). ChIP‒qPCR showed that NFE2L3 could indeed bind the promoter region of the LASP1 transcript to regulate it (Fig. 7e). The binding sites of NFE2L3 and LASP1 transcripts mainly occurred in the common sequences of GTCACWCT (W = T or A or C or G) (Fig. 7f). Dual luciferase reporter experiments showed that NFE2L3 could regulate the LASP1 promoter region, and the fluorescence intensity was significantly reduced after mutation in the binding region, indicating that NFE2L3 could promote the expression of LASP1 (Fig. 7g). It has been reported that LASP1 can activate the AKT/GSK3β pathway [20], and the AKT/GSK3β pathway is also activated in ccRCC [21]. Therefore, we examined whether NFE2L3 promotes the progression of ccRCC by regulating LASP1 and thereby activating the AKT/GSK3β pathway. Our results showed that after NFE2L3 knockdown, LASP1 expression was decreased, phosphorylation of AKT and GSK3β was inhibited, and β-catenin expression was also decreased (Fig. 7h), as was also the case after NAT10 knockdown (Fig. 7i). We wanted to confirm that NAT10 indeed regulates the expression of LASP1 and the activation of the AKT/GSK3β pathway by regulating the expression of NFE2L3, and the results showed that the expression of LASP1 and phosphorylation of AKT/GSK3β were not changed after NAT10 knockdown in NFE2L3-knockdown cells (Fig. 7j), the consistent results were obtained by knocking down HIF-1α under normoxic and hypoxic conditions (Supplementary Fig. S4d). Therefore, NAT10 indeed regulates the expression of LASP1 and activation of the AKT/GSK3β pathway by regulating the expression of NFE2L3. The TCGA database showed that the expression of LASP1 was increased in ccRCC tissues (Fig. 7k), which was consistent with the effect of NAT10 and NFE2L3. The expression of NFE2L3 in ccRCC tissues is positively correlated with the expression of LASP1 (Fig. 7l). Overexpression of NFE2L3 or LASP1 after NAT10 knockdown can restore the changes of cell migration caused by NAT10 knockdown (Supplementary Fig. S5a). In conclusion, NAT10 regulates the expression of LASP1 by regulating NFE2L3, thus activating the AKT/GSK3β pathway to promote the progression of ccRCC. We also obtained the consistent results in HeLa cell and gastric cancer cell MGC-803. Knockdown NFE2L3 or LASP1 restored the changes in cell proliferation and migration caused by overexpression of NAT10, indicating that NAT10 might perform similar functions through NFE2L3/LASP1 signalling in other cancers (Supplementary Fig. S5b–e).

## Discussion

RNA modification plays an important role in many diseases, including tumours. Ac4C RNA modification is a newly discovered modification mode on a variety of RNAs, and at present, only NAT10 has been found to modify ac4C [9]. Recently, in addition to its effect on mRNA, NAT10-mediated ac4C modification has also been found to play an important role in tumours. It has been reported that mRNA ac4C acetylation plays an important role in cancers such as gastric cancer, bladder cancer and multiple myeloma [7, 16, 22], although its role in ccRCC has not been reported. Currently, first-line therapy, which mainly targeting VEGF and mTOR, is used for advanced ccRCC [23]. Effective targeted therapy is still limited, and therefore it is very important to find new targeted molecules for the diagnosis and treatment of ccRCC. Our study found that NAT10-mediated ac4C modification of NEF2L3 mRNA promoted the occurrence and development of ccRCC.

Studies have found that NAT10 has lysine acetylase activity and is involved in DNA damage repair through protein acetylation [24–26] and activation of hTERT to activate telomerase activity [27]. Recently, it has also been shown that NAT10-mediated mRNA ac4C modification plays an important role in tumour development. Our study confirms the clinical significance of NAT10 in ccRCC. Immunohistochemical staining of ccRCC tissues and adjacent tissues showed that the expression of NAT10 increased in ccRCC and was correlated with the grade of ccRCC. TCGA database analysis also showed that NAT10 expression was upregulated in ccRCC. Kaplan‒Meier survival analysis showed that NAT10 was associated with a poor prognosis in patients with ccRCC. This is consistent with the results of previous studies on the high expression of NAT10 in bladder cancer [7], gastric cancer [16], pancreatic cancer [28] and other tumours. Moreover, through knockdown and overexpression of NAT10, we found that NAT10 promoted cell proliferation and migration and cell cycle progression. In vivo, the results of subcutaneous tumour formation and caudal vein metastasis in mice showed that knockdown of NAT10 inhibited tumour growth and metastasis and that intraperitoneal injection of Remodelin effectively inhibited tumour growth. In summary, NAT10 is associated with poor prognosis in patients with ccRCC and may be used as a biomarker for the occurrence and progression of ccRCC, as well as a potential target for future clinical diagnosis and treatment of ccRCC.

Clear cell renal cell carcinoma often has mutations in VHL tumour suppressor genes, leading to upregulation of the HIF family. HIF-1α is a major regulator of hypoxia-induced gene transcriptional activation [29]. Our study found that HIF-1α upregulates the expression of NAT10 and promotes the progression of ccRCC. However, the regulation of NAT10 by other HIF family molecules was not investigated in this paper. Our predictions using the PROMO website showed that only HIF-1α in the HIF family regulated the NAT10 promoter, and therefore we did not assess the effects of other HIF family molecules on NAT10. Other HIF family molecules could be knocked down or knocked out in ccRCC cell lines to detect their effects on NAT10, and ChIP‒qPCR could be used for verification in future studies.

To clarify the mechanism by which NAT10 promotes proliferation and metastasis in ccRCC, we screened downstream target molecules of NAT10 by acRIP-seq, RNA-seq, acRIP and RIP and found that NAT10 could bind NFE2L3 mRNA to induce ac4C acetylation modification. Studies by Arango have shown that ac4C acetylation mainly occurs in the CDS region of mRNA [9]. In our study, the ac4C modification site of NFE2L3 mRNA was indeed in the CDS region, which is consistent with the research results. After mutation of the ac4C modification site of NFE2L3 mRNA, knocking down NAT10 did not affect the expression of NFE2L3. Therefore, we believe that NAT10 primarily affects the expression of NFE2L3 by mediating ac4C modification of NFE2L3 mRNA rather than affecting its protein acetylation. As a transcription factor, NFE2L3 can enhance the expression of POMP, which enhances the assembly and activity of the 20S proteasome, thereby degrading the tumour suppressor proteins p53 and Rb in an ubiquitin-independent manner and increasing cell death resistance and cell cycle arrest in cells [30]. NFE2L3 is highly expressed in Hodgkin’s lymphoma, pancreatic cancer, hepatocellular carcinoma, gastric cancer, colorectal cancer and other malignant tumours [31–37]. By analyzing the ChIP-seq and RNA-seq results, LASP1, with the highest expression, was selected among the candidate genes, and it was confirmed that NFE2L3 could indeed regulate the expression of LASP1, activating the AKT/GSK3β pathway [20] and promoting cell proliferation and migration. Y-G Zhang believed that in addition to NAT10, there might be other mRNA ac4C “writer” proteins in the ac4C acetylation modification of mRNA, similar to m6A, m5C and m1A [38–40], and there might also be “eraser” proteins, which served as a dynamic process of joint regulation [16]. In this paper we did not explore the effects of other potential mRNA ac4C modification proteins. The results of acRIP-seq and RNA-seq showed that NAT10 could regulate multiple pathways. We found that NFE2L3 was the main downstream molecule of NAT10 through preliminary experiments. Overexpression of NFE2L3 or LASP1 could basically restore the effect of NAT10 knockdown on cell function, but not completely, which suggested that NAT10 might exert its influence on ccRCC by affecting other signalling pathways. We noticed that NAT10 could affect tumour-related signalling pathways by analysis of the sequencing data. Moreover, NAT10 promotes gastric cancer by regulating COL5A1 [16], and promotes bladder cancer progression by participating in DNA damage repair pathways [41]. It suggested that NAT10 could exert tumour-promoting effects through multiple pathways. In addition, in this study, we mainly focused on the effect of NAT10-mediated ac4C modification on mRNA stability, while mRNA ac4C modification may also affect the translational efficiency of mRNA [9]. Our study did not prove that NAT10 could regulate translation efficiency of NFE2L3 directly by regulating ac4C modification of NFE2L3 mRNA, which could be verified by Ribo-seq technique in subsequent studies. Therefore, it is also possible that NAT10 might affect the progression of ccRCC by regulating other downstream molecules, which needs to be explored in the future studies.

In summary, our findings suggest that one of the cancer-promoting effects of NAT10 is to promote the expression of NFE2L3, form the NAT10-NFE2L3-LASP1-Akt/GSK3β axis, and promote the occurrence and progression of ccRCC. This paper also shows that NAT10 small molecule inhibitors can inhibit tumour proliferation, and therefore the targeted inhibition of NAT10 may be a potential treatment for ccRCC in the future.

## Materials and methods

### Tissue specimens

The cancer and paracancerous tissues of ccRCC patients were provided by Qilu Hospital of Shandong University and preserved in liquid nitrogen. Informed consent of the patients was obtained, and the Ethics Committee of Shandong University Qilu Hospital approved the study after obtaining informed consent from all participants in accordance with the provisions of the 1975 Declaration of Helsinki.

### Cell culture and treatment

The human ccRCC cell lines 786-O and A498 were purchased from the Institute of Basic Medicine, Chinese Academy of Medical Sciences. 786-O cells were cultured in 1640 medium with 10% foetal bovine serum, and A498 cells were cultured in DMEM with 10% foetal bovine serum in a 5% CO_2_ cell incubator at a constant temperature of 37 °C. The jetPRIME transfection reagent was used to transfect siRNAs and plasmids into the cells. The siRNA sequences are shown in Supplementary Table S2. The NAT10 plasmid was synthesized by Scientific Yun Company, and the NFE2L3-3×Flag plasmid was purchased from MiaoLing Plasmid Platform. The lentivirus and lentivirus control with NAT10 knockdown were constructed by Shanghai Jikai Company.

### Cell proliferation assay

Cell proliferation was assessed using Cell Counting Kit-8 (CCK8, APEXBIO) reagents. Then, after transfection of plasmid or siRNAs for 48 h, the cells were plated in 96-well plates at 3×10^3^ cells/well, with five replicate wells used for each treatment. The detection reagent was added according to the specified time and incubated in the incubator for 1 hour and 20 minutes. The absorption at a 450 nm wavelength of the reaction products was determined by an enzyme labelling instrument.

### EdU experiment

After transfection of plasmids or siRNAs for 48 h, the cells were plated at 3×10^3^ cells/well in 96-well plates, with three replicate wells used for each treatment. After 16 h, a BeyoClickTMEDU-488 cell proliferation detection kit was used.

### Colony formation assay

Forty-eight hours after transfection of plasmid or siRNAs, 2 ml complete medium was added to each well of the 6-well plate with a cell density of 1 500 cells per well. After 10 days of cell culture, the cells were fixed with 4% paraformaldehyde and stained with 0.5% crystal violet, and the cell clones were counted under a microscope. Only cell masses containing more than 50 cells were considered cell clones.

### Flow cytometric analysis

After transfection of plasmids or siRNAs for 48 h, the cells were collected and treated with PI/RNase buffers. A Cytoflex instrument was used for detection. The percentage of cells at different stages of the cell cycle was analyzed using FlowJo software.

### Cell migration experiment

The Transwell chamber was placed in the medium, and the cell suspension after transfection with plasmid or siRNAs for 48 h was added into the chamber at a cell density of 1.5 × 10^4^ cells/well. The cells were placed in the cell incubator for 36–48 h, fixed with 4% paraformaldehyde and stained with 0.5% crystal violet. The membrane was dried and counted using a 200× microscope. The number of migrating cells was represented by three randomly selected field cells.

### Quantitative RT‒PCR

RNA was extracted using Sparkjade’s SPARKeasy Cell RNA Rapid Extraction Kit, RNA concentration was detected using a Nanodrop One ultrafine spectrophotometer, and cDNA was converted using a Vazyme reverse transcription reagent. Real-time quantitative PCR detection was performed using ChamQ SYBP QPCR Master Mix, and the sequence is shown in Table S2.

### Western blotting

The protein extracts were separated by 8–15% SDS‒PAGE gel and wet-transferred to a PVDF membrane for detection by a primary antibody and a peroxidase-conjugated anti-mouse or -rabbit antibody (Absin) as a secondary antibody. The antigen-antibody reaction was observed by enhanced chemiluminescence. The antibodies against the following proteins were used: Anti-NAT10 (ab194297, Abcam), NFE2L3 (A15761, ABclonal), HIF1 alpha (340462, ZENBIO), GAPDH (abs132004, Absin), Flag (F1804, Sigma), LASP1 (10515-1-AP, Proteintech), AKT (YT0185, Immunoway), pAKT (YP0006, Immunoway), GSK3β (GB11099, Servicebio), pGSK3β (YP0124, Immunoway), and β- catenin (8480S, CST).

### RNA decay

The cells were treated with actinomycin D at a final concentration of 2 μM for 0 h, 6 h, and 12 h. Total RNA was extracted, and the relative level of NFE2L3 mRNA was determined by real-time quantitative PCR. The half-life of the NFE2L3 mRNA was estimated. In this study, GAPDH mRNA was used as an endogenous control.

### Dual luciferase assay and translation efficiency

After constructing NAT10 knockdown cells, cDNA containing NFE2L3 acetylated fragments was cloned into the pmirGLO vector. The mutant plasmid replaced C with A in the CCXCCXCC ac4C sequence on the mRNA of the acetylated fragment of NFE2L3, and the inserted sequence is shown in Table S3. The wild-type and mutant reporter plasmids were transfected into 786-O-shNC, 786-O-shNAT10, A498-shNC and A498-shNAT10 cells. After 48 h, the cells were collected, and luciferase activity was assessed on a Varioskan LUX multifunctional enzyme marker. We defined mRNA translation efficiency as relative fluorescence intensity/relative RNA expression [16].

### acRIP and acRIP-seq

The acRIP analysis was performed in the NAT10 control group and NAT10 knockdown group using the GenSeq® ac4C RIP kit from Cloud-Seq Biotech Co.,LTD (shanghai, China). Total RNA (200 µg) was randomly digested into 200 nt chains, and a mixture of 2 µg ac4C antibodies and magnetic beads was incubated with the fragmented RNA. The RNA was then purified and analyzed by RT‒qPCR. The acRIP-qPCR primers are shown in Table S2. Four pairs of ccRCC cancer tissues and their corresponding paracancerous tissues were used for acRIP-seq. acRIP and acRIP-seq were performed by Cloud-Seq Biotech Co.,LTD(shanghai,China) (See Supplementary material 2 for detailed analysis).

### RIP

The Magna RIPTM RNA-binding protein immunoprecipitation kit was used for the RIP-qPCR assay, and the RIP assay was performed in MKN-45 cells to evaluate and verify the interaction between NAT10 and NFE2L3 mRNA. Anti-rabbit immunoglobulin G (5 µg) or NAT10 (ab194297, Abcam) antibodies were bound to magnetic beads and incubated overnight at 4 °C with prefrozen cell lysates (more than 1 × 10^7^ cells per sample). Immunoprecipitates containing RNA‒protein complexes were digested by protease K, and RNA was extracted using phenol:chloroform:isoamyl alcohol (125:24:1 pH = 4.3). Subsequently, the expression level of NFE2L3 mRNA was determined by RT‒qPCR. The primers used for RIP-qPCR are shown in Table S2.

### ChIP and ChIP-seq

The CST SimpleChIP® Enzymatic Chromatin IP Kit (Magnetic Beads) (9003S) was used. Cells were cross-linked with 1% formaldehyde at room temperature for 10 min. After washing with cold PBS, cells were collected and lysed with nuclease for 20 min. The length of the DNA in the lysate was between 150 and 900 bp. The immune complex was then washed three times (5 minutes/wash) with a low-salt solution and once with a high-salt solution. The cross-linked protein was immunoprecipitated with a HIF-1α antibody (ab243860) or a nonspecific IgG antibody (2729, CST). The chromatin was eluted from the immune complex by heating at 65 °C for 30 min. After digestion with protease k, DNA was purified by a centrifuge column for ChIP‒qPCR. After transfection of the pcDNA3.4-NFE2L3-3×Flag plasmid into 786-O cells for 48 h, cross-linked protein (NFE2L3) was immunoprecipitated with a mouse anti-human Flag monoclonal antibody (F1804, Sigma) or a nonspecific IgG antibody (2729, CST). DNA was purified, and ChIP-seq was performed. ChIP-seq was performed by SEQHEALTH. All primer sequences are shown in Table S2(See Supplementary material 2 for detailed analysis).

### RNA-seq

Total RNA was extracted by TRIzol (RNA > 2 μg/group). Eukaryotic mRNA was enriched by magnetic beads with Oligo(dT). mRNA was fragmented into fragments by fragmentation buffer, and cDNA was synthesized with six-base random primers. Then, the buffer solution, dNTPs and DNA polymerase I were added to synthesize the double-stranded cDNA. The eluted and purified double-stranded cDNA was then end-repaired, base A was added, and the sequencing joint was added. The 5’ end of the cDNA was connected to the UID joint, and the target size fragments were recovered by magnetic beads and amplified by PCR. An Illumina sequencing instrument was used for high-throughput sequencing after a quality control inspection. RNA-seq was completed by SEQHEALTH (See Supplementary material 2 for detailed analysis).

### Immunohistochemistry staining

Immunohistochemical staining was performed with an immunohistochemical staining kit (ZSBG-BIO), a panoramic digital section microscope was used for imaging.

### Animal experiment

The effects of NAT10 on tumorigenesis in BALB/c female nude mice aged 4 weeks were analyzed. Eighteen mice were randomly divided into three groups. A498-shNAT10 cells and A498-shNC cells (5 × 10^6^/100 µl per mouse, n = 6 per group) were collected and suspended in precooled PBS and injected subcutaneously into mice. The change in tumour volume was closely monitored (volume = width^2^ × length/2). After the subcutaneous tumour volume of A498-shNC cells reached 50 mm^3^, the NAT10 inhibitor Remodelin was injected intraperitoneally. After 66 days, the mice were sacrificed, and the tumours were removed.

To investigate the effect of NAT10 on metastasis, 14 mice in BALB/c nude mice aged 4 weeks were randomly divided into two groups. Each mouse was injected with A498-shNAT10 cells and A498-shNC cells (5 × 10^6^/120 µl) via the caudal vein. After 32 days, in vivo imaging was performed on mice, and the liver and lung were collected for liver and lung metastasis nodule counts and HE staining. This study was approved and guided by the Ethics Committee of the Shandong University Qilu Hospital.

### Statistical analysis

Statistics were performed using GraphPad Prism 8.0. Data are from at least three independent trials. When more than two groups were compared, ANOVA was used for statistical analysis, and Student’s *t*-test was used for comparisons between two groups. For continuous variables with nonnormal distributions, the nonparametric Kruskal‒Wallis rank sum test was used. Kaplan‒Meier survival curves were compared using the logarithmic rank test. The differences were considered statistically significant when *P < 0.05, **P < 0.01, or ***P < 0.001.

#### Date deposition

The acRIP-seq,RNA-seq and ChIP-seq sequencing data used in this study has been uploaded to the Gene Expression Omnibus (GEO) with the accession code GSE226863, GSE226941 and GSE226940.

## Supplementary information

Original Data

Supplemental Material

## Data Availability

All data are available in this manuscript and supplementary files.
