# Supplementary material for: NAT10 promotes the progression of clear cell renal cell carcinoma by regulating ac4C acetylation of NFE2L3 and activating AKT/GSK3β signaling pathway
Source: Cell Death Dis. 2025 Apr 2;16(1):235. doi: 10.1038/s41419-025-07528-w (PMC11962090; doi:10.1038/s41419-025-07528-w)

Supplemental Material-western blot-1

Fig.2b

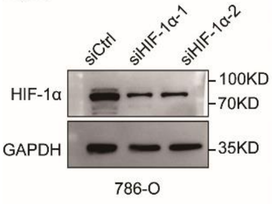

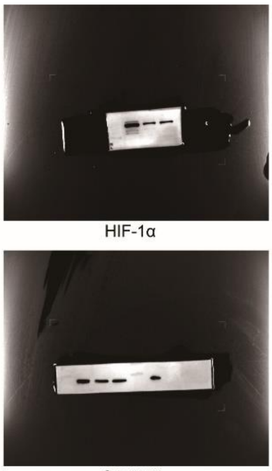
HIF-1α

GAPDH

Fig.3a

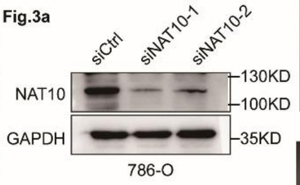

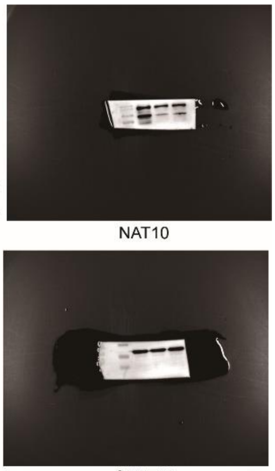
NAT10

GAPDH

Fig.2d

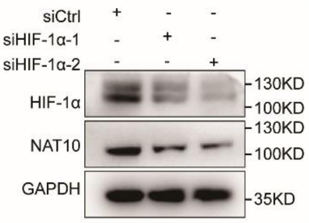

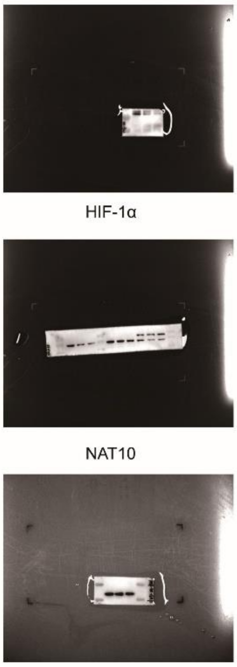
HIF-1α

NAT10

GAPDH

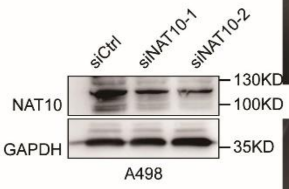

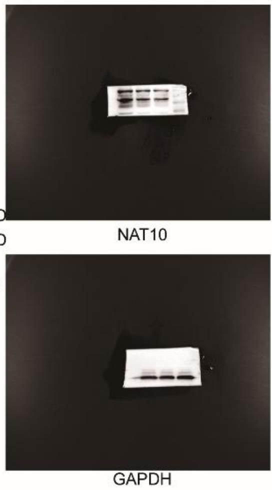
NAT10

GAPDH

Supplemental Material-western blot-2

Fig.3j

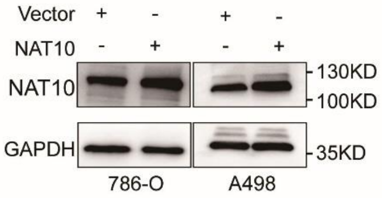

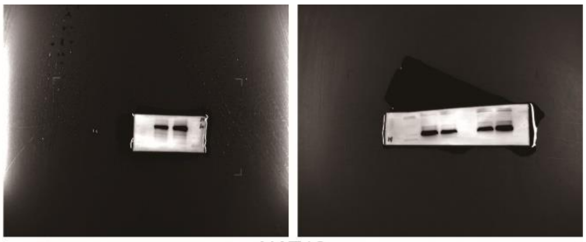

NAT10

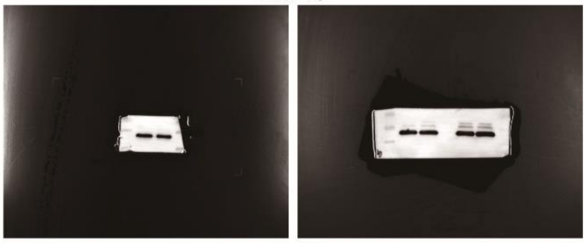

GAPDH

Fig.4b

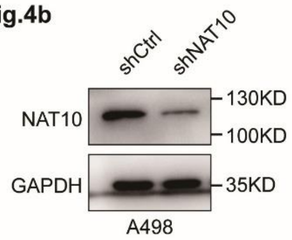

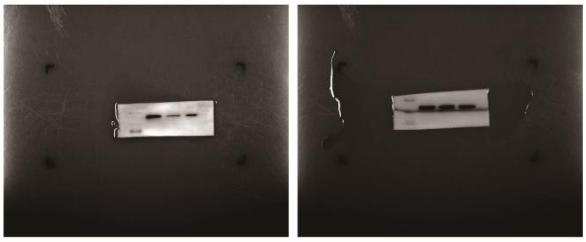

NAT10 GAPDH

Fig.5i

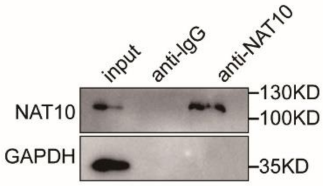

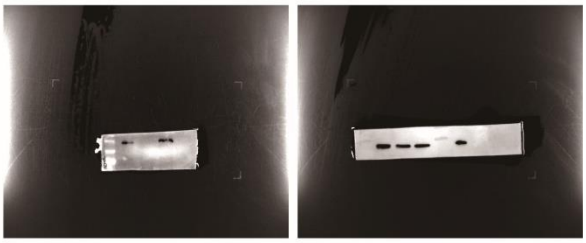

NAT10 GAPDH

Fig.5k

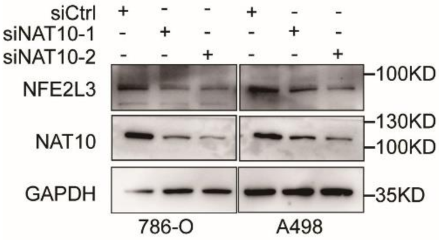

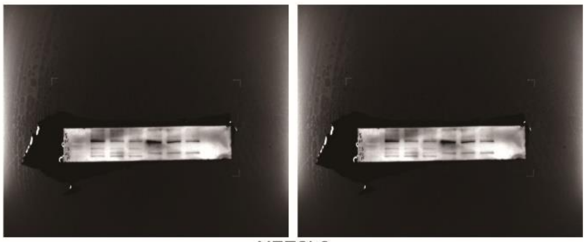

NFE2L3

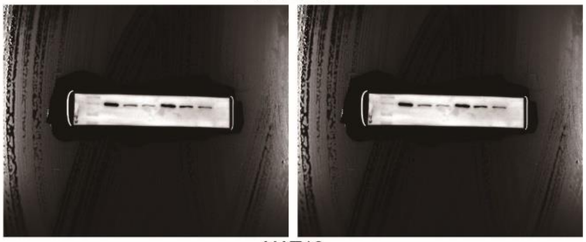

NAT10

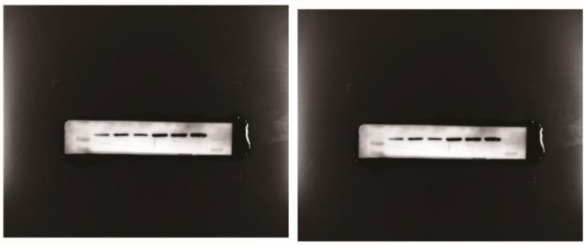

GAPDH

Supplemental Material-western blot-3

Supplemental Material 2 g

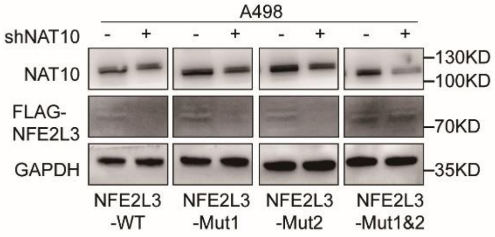

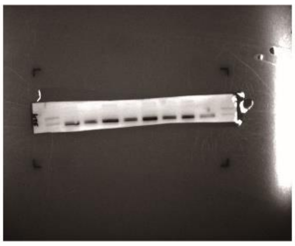
NAT10

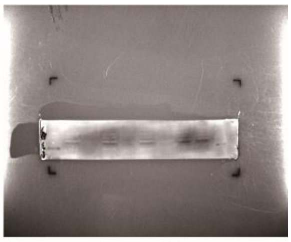
FLAG-NFE2L3

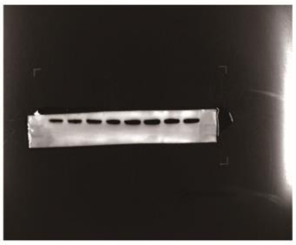
GAPDH

Supplemental Material 2 h

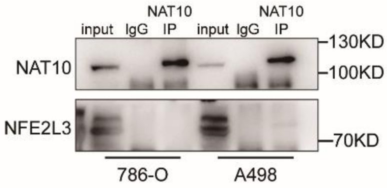

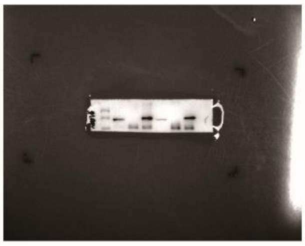
NAT10

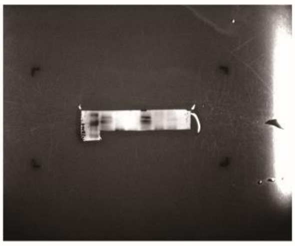
NFE2L3

Fig.6d

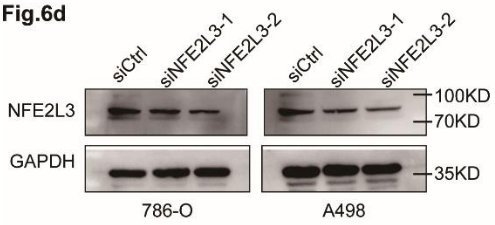

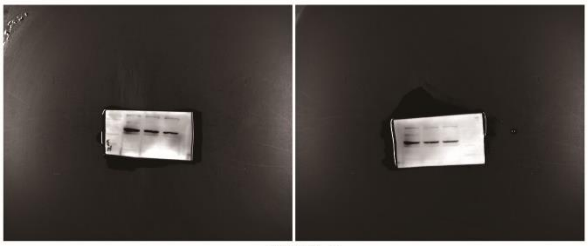
NFE2L3

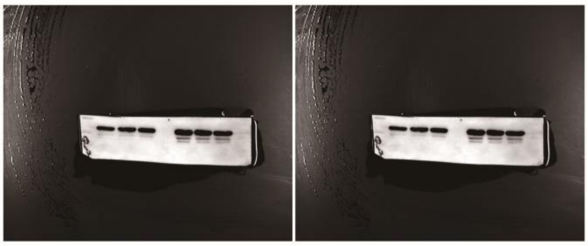
GAPDH

Supplemental Material-western blot-4

Fig.7h

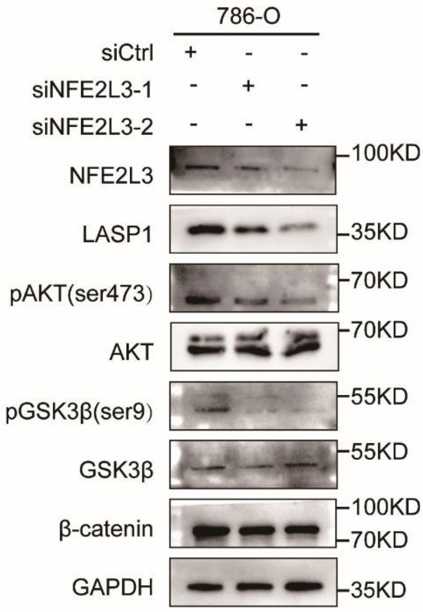

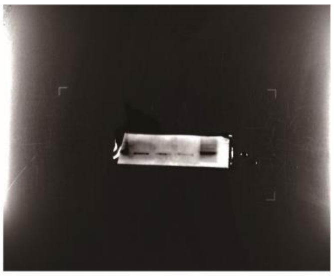
NFE2L3

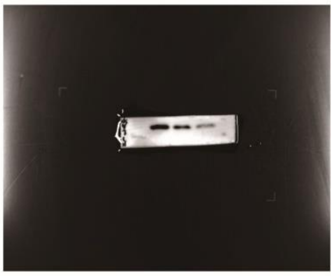
LASP1

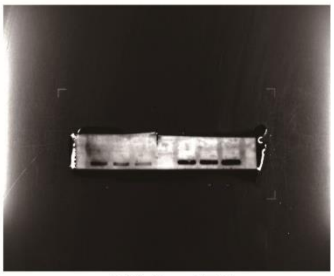
pAKT(ser473)

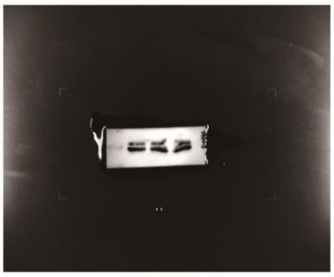
AKT

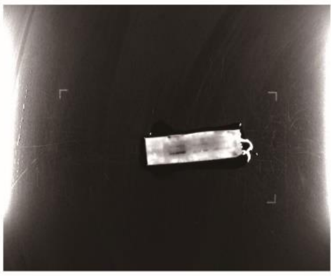
pGSK3β(ser9)

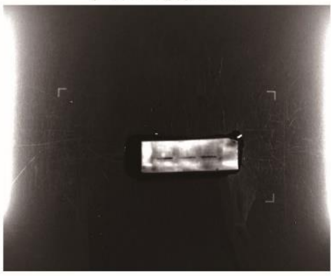
GSK3β

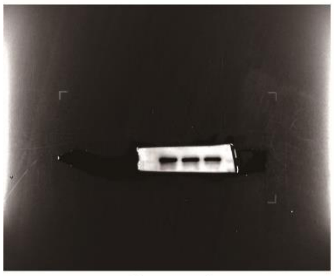
β-catenin

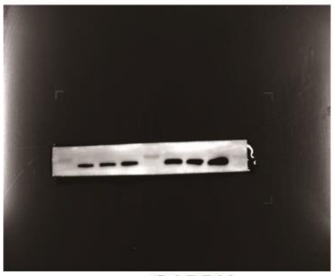
GAPDH

Supplemental Material-western blot-5

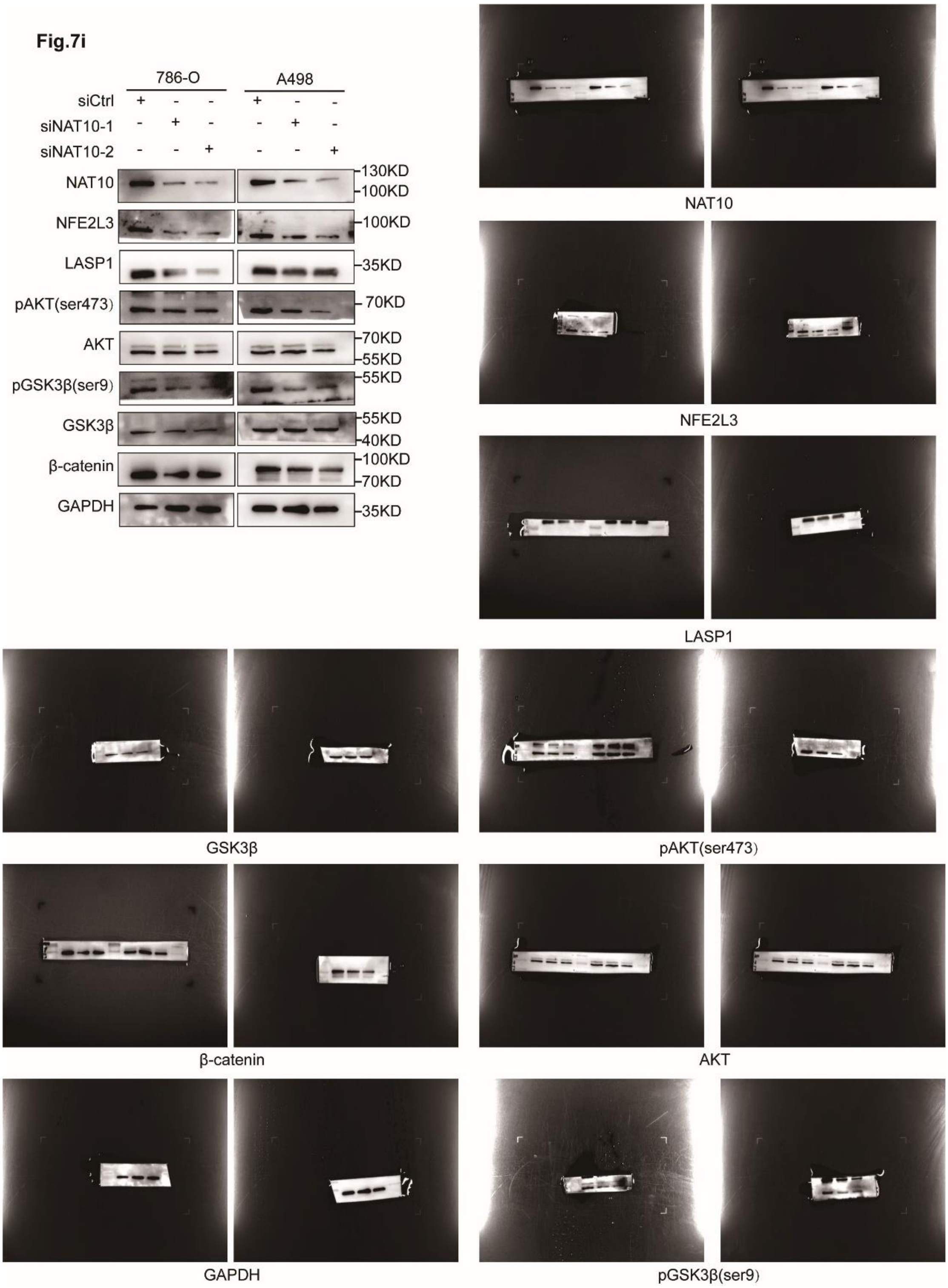

Supplemental Material-western blot-6

Fig.7j

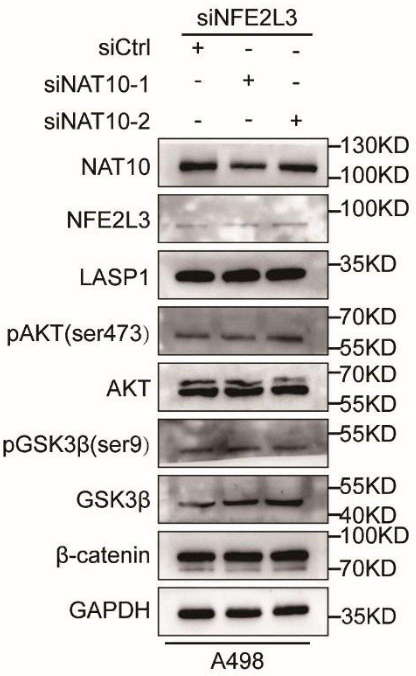

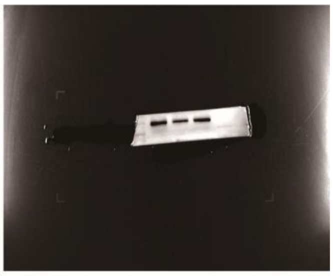

NAT10

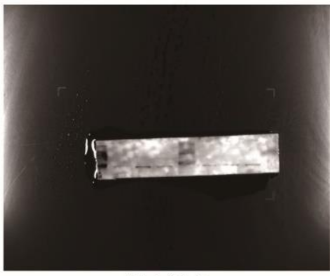

NFE2L3

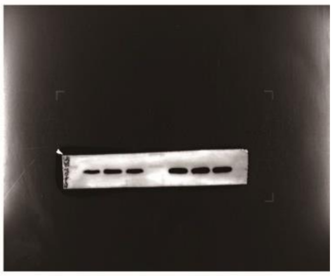

LASP1

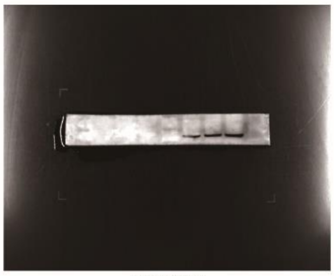

GSK3β

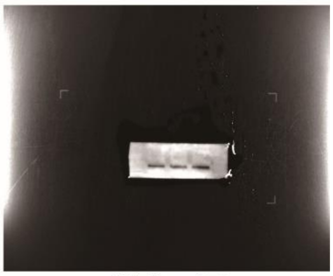

pAKT(ser473)

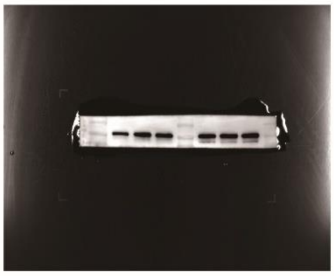

β-catenin

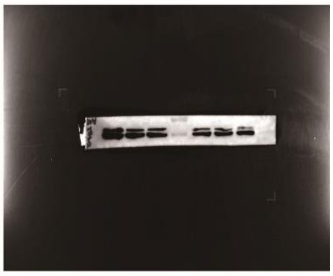

AKT

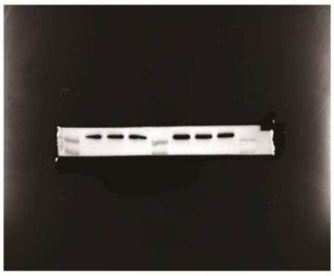

GAPDH

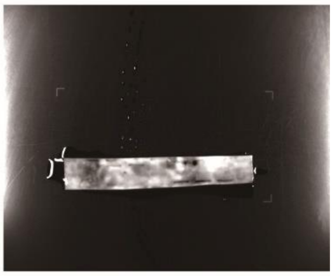

pGSK3β(ser9)

Supplemental Material-western blot-7

Fig.5o

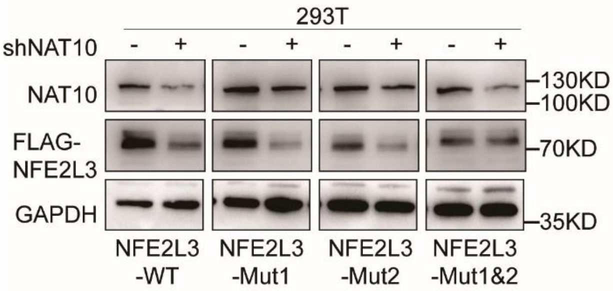

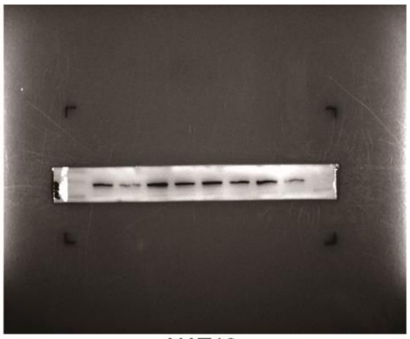

NAT10

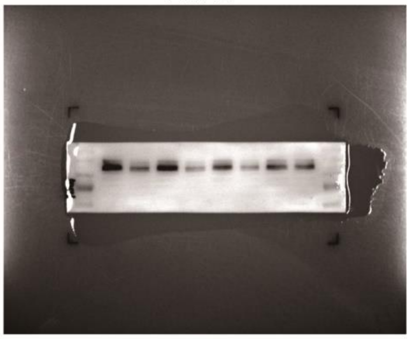

FLAG-NFE2L3

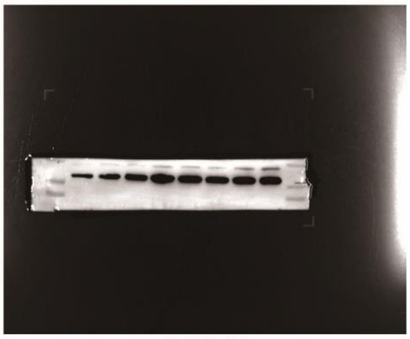

GAPDH

Supplemental Material-western blot-s4

Fig.S4d

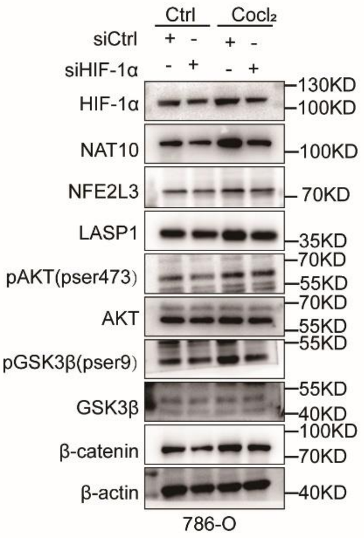

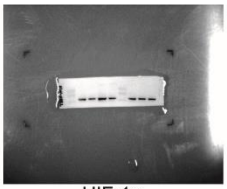

HIF-1α

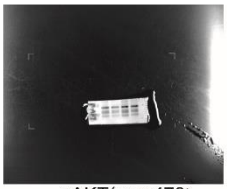

pAKT(pser473)

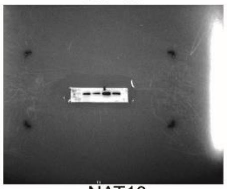

NAT10

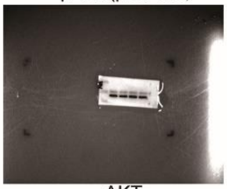

AKT

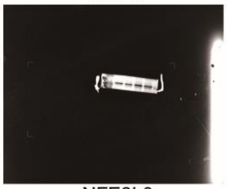

NFE2L3

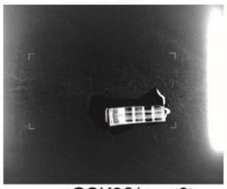

pGSK3β(pser9)

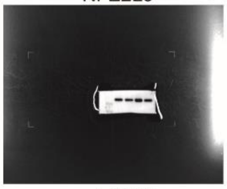

LASP1

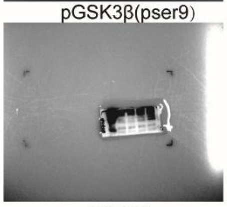

GSK3β

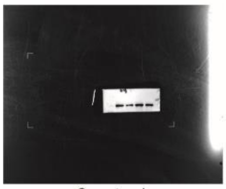

β-catenin

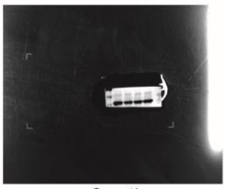

β-actin

Fig.S1j

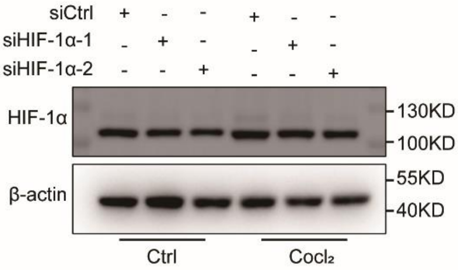

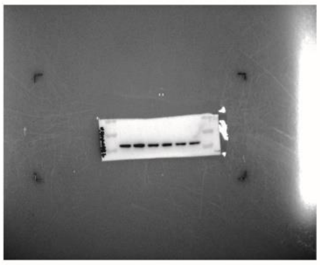

β-actin

Hif-α

Supplemental Material-western blot-s5

Supplementary Fig5a

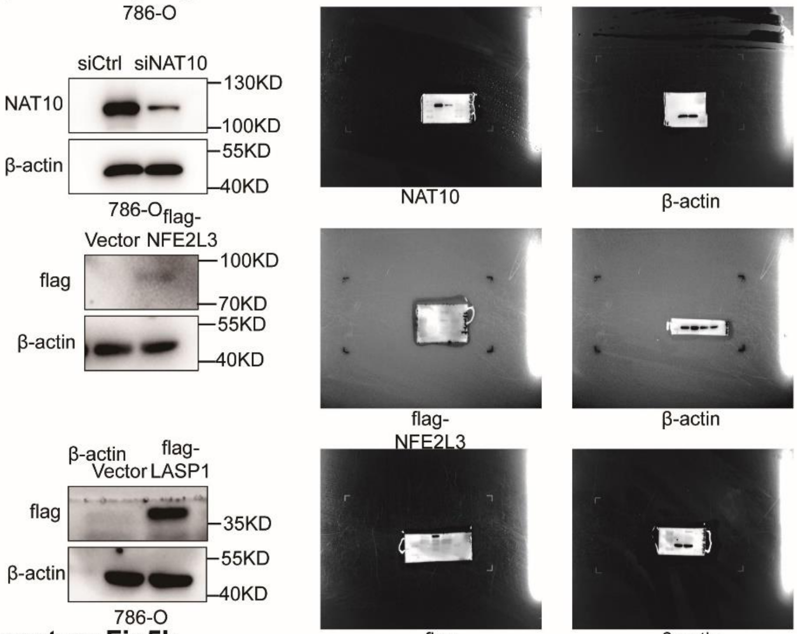

Supplementary Fig5b

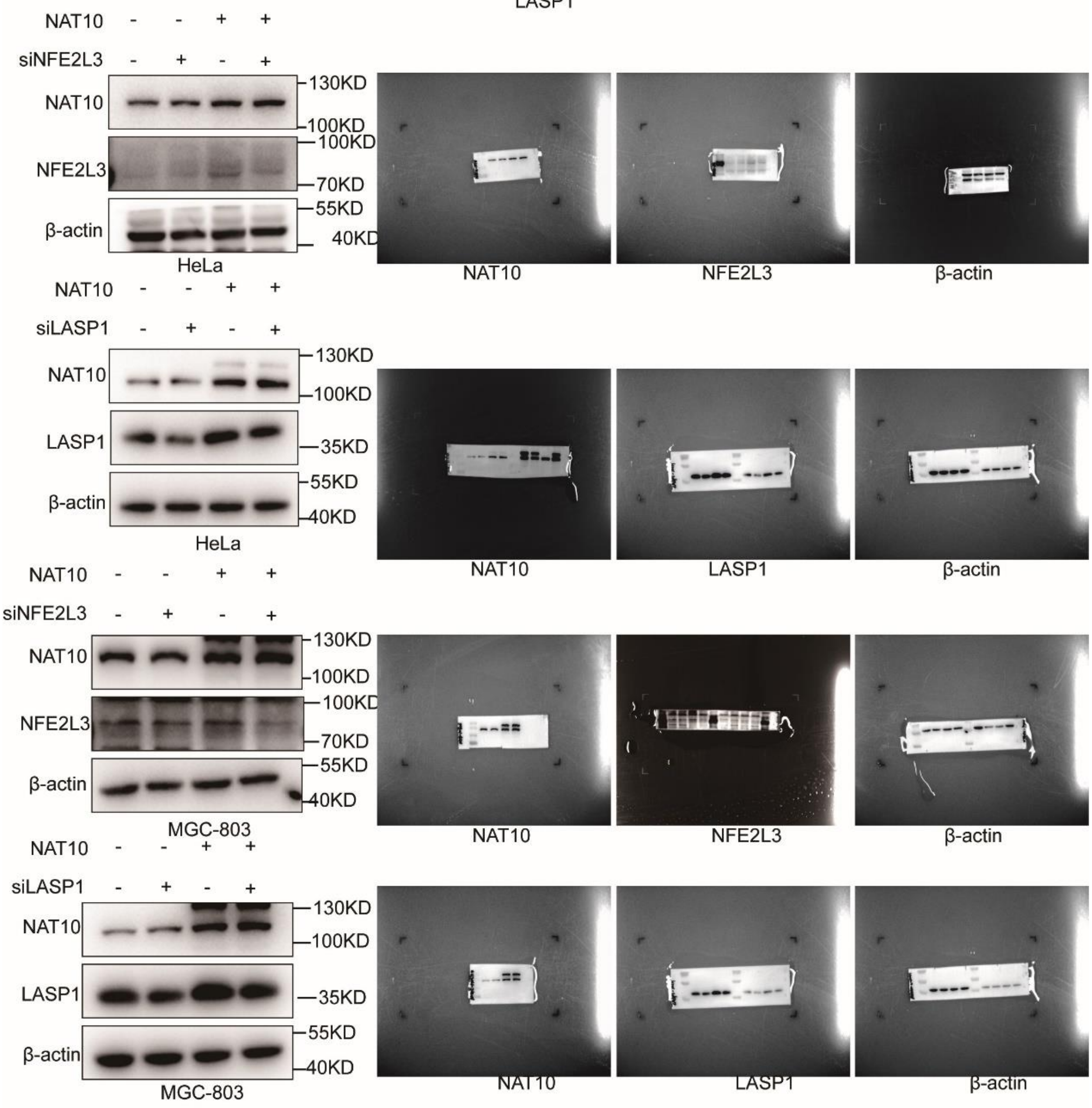

Supplement: Supplementary file 1 — Original Data [file 41419_2025_7528_MOESM1_ESM.pdf]
