# Supplementary material for: NAT10 promotes the progression of clear cell renal cell carcinoma by regulating ac4C acetylation of NFE2L3 and activating AKT/GSK3β signaling pathway
Source: Cell Death Dis. 2025 Apr 2;16(1):235. doi: 10.1038/s41419-025-07528-w (PMC11962090; doi:10.1038/s41419-025-07528-w)
Supplement: Supplementary file 2 — Supplemental Material [file 41419_2025_7528_MOESM2_ESM.pdf]

Supplementary figure S1

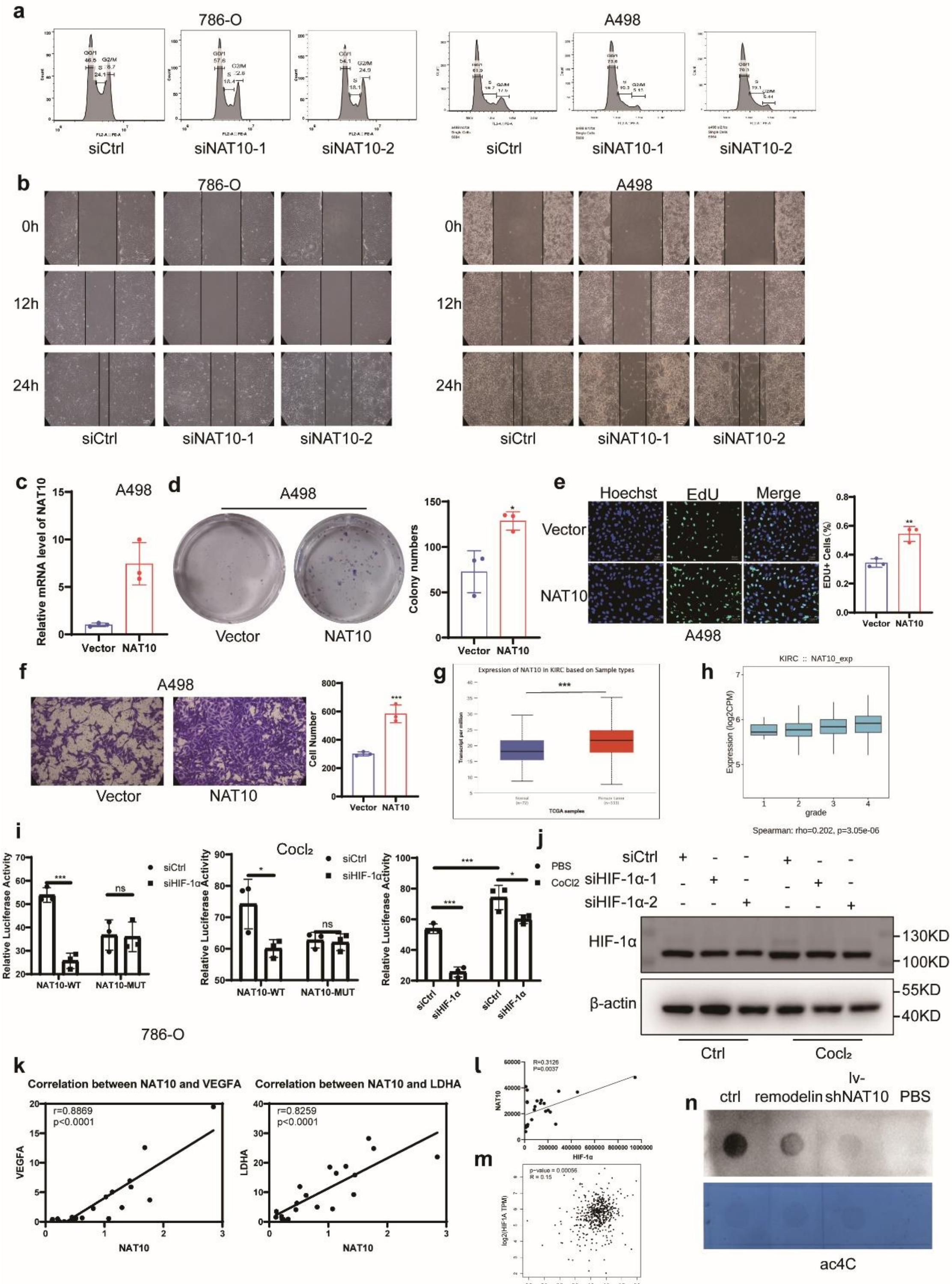

Supplementary figure S2

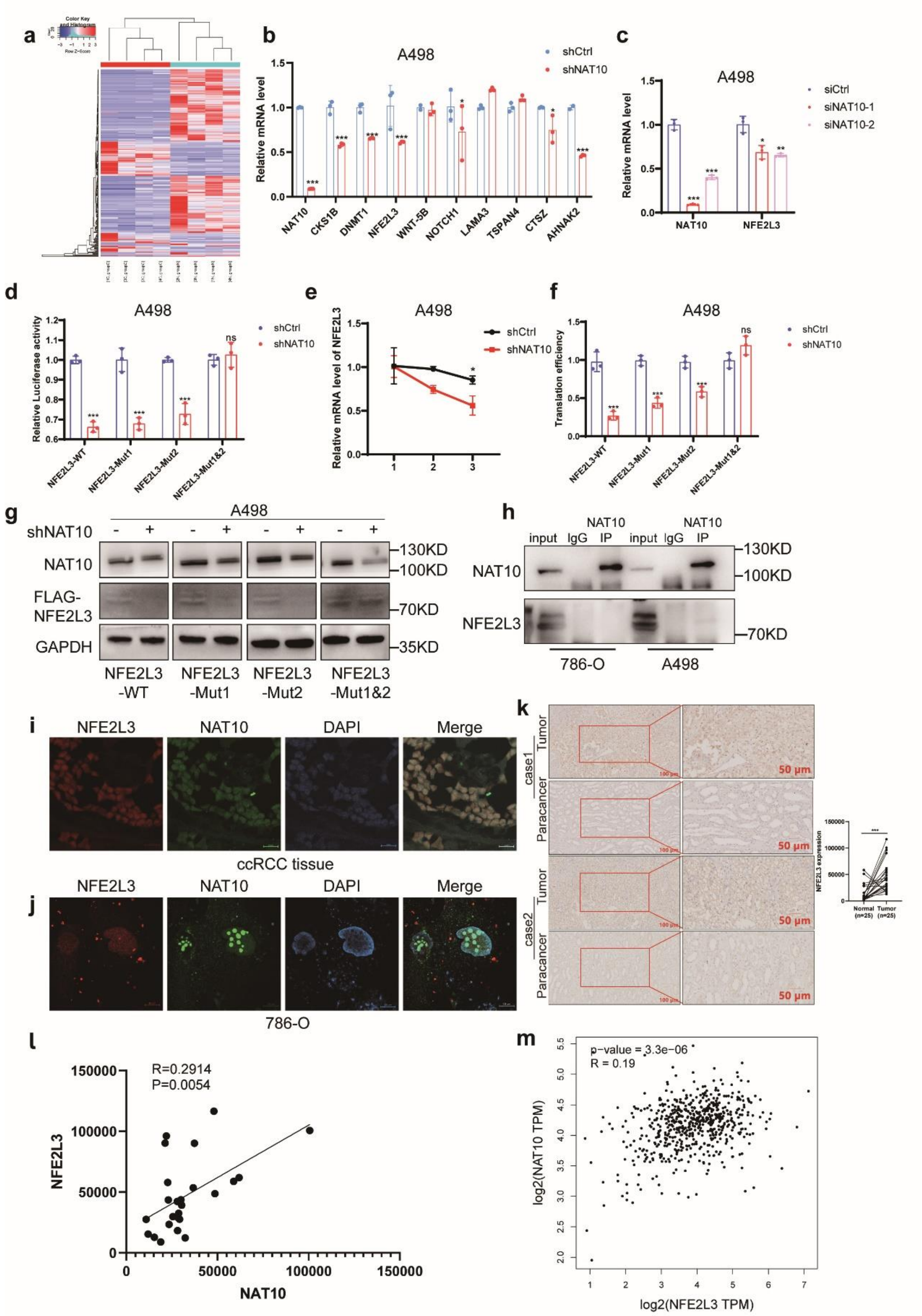

Supplementary figure S3

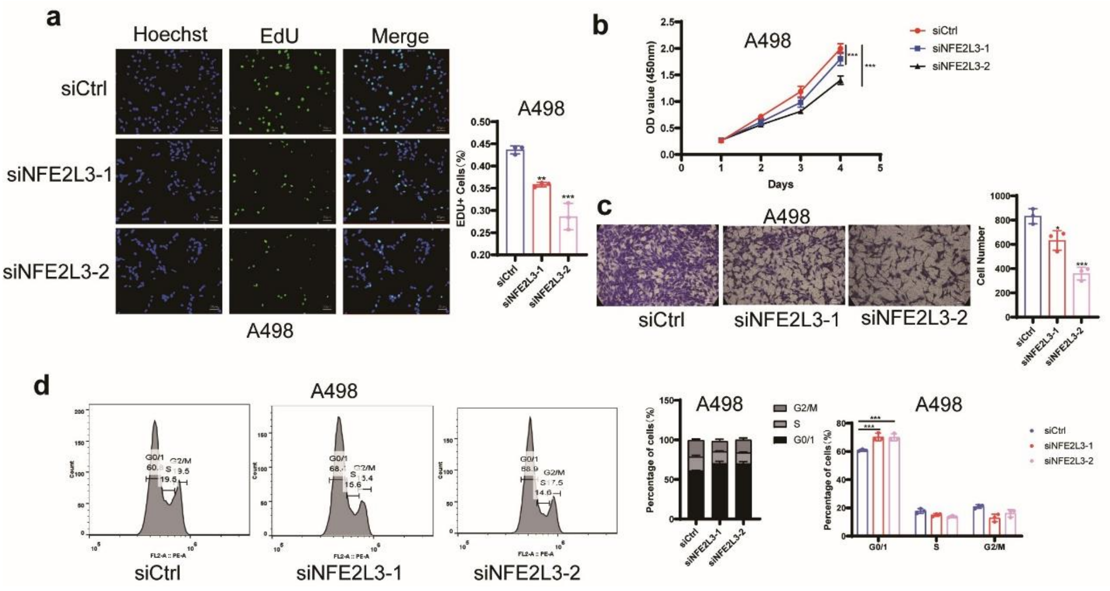

Supplementary figure S4

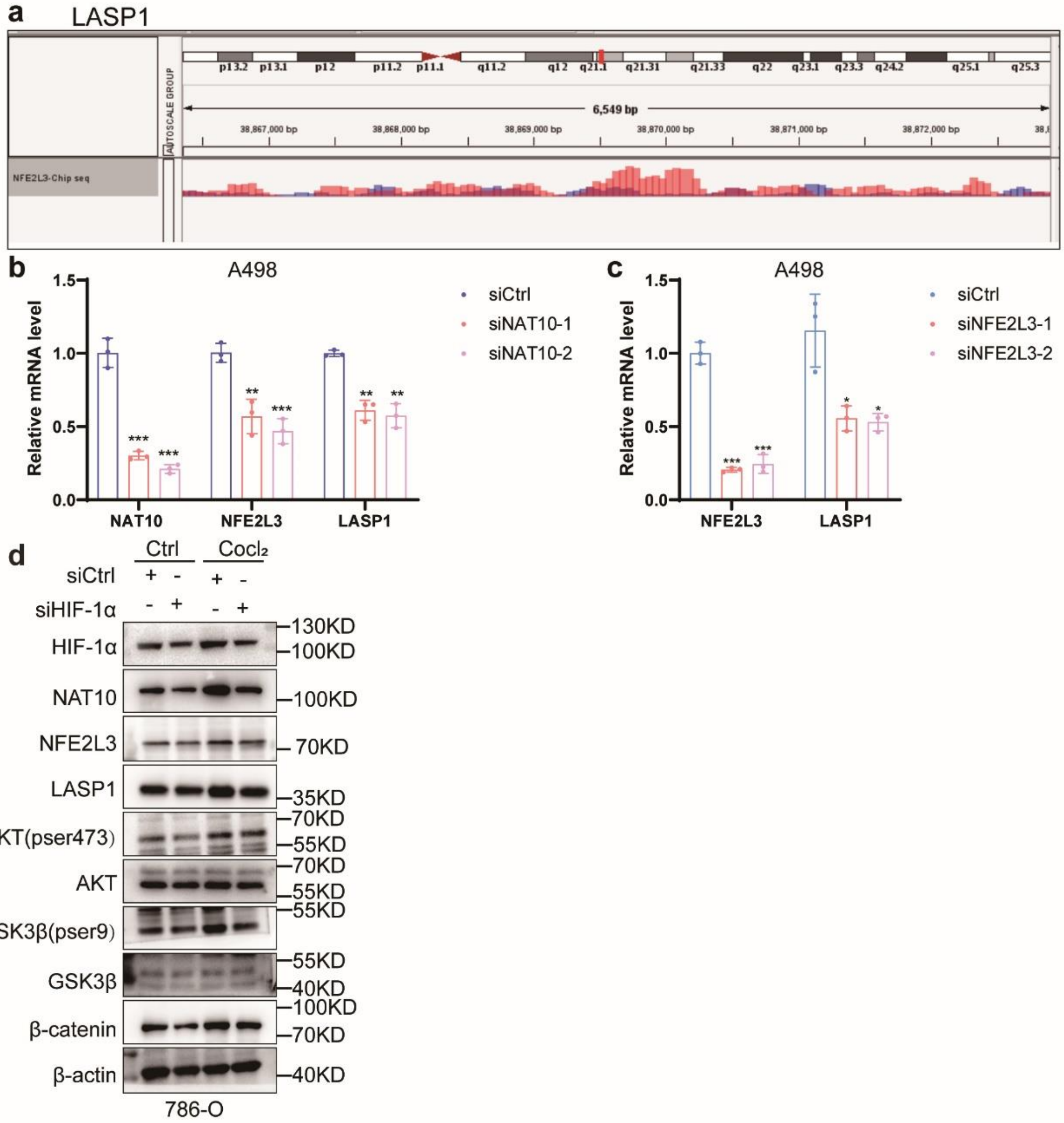

Supplementary figure S5

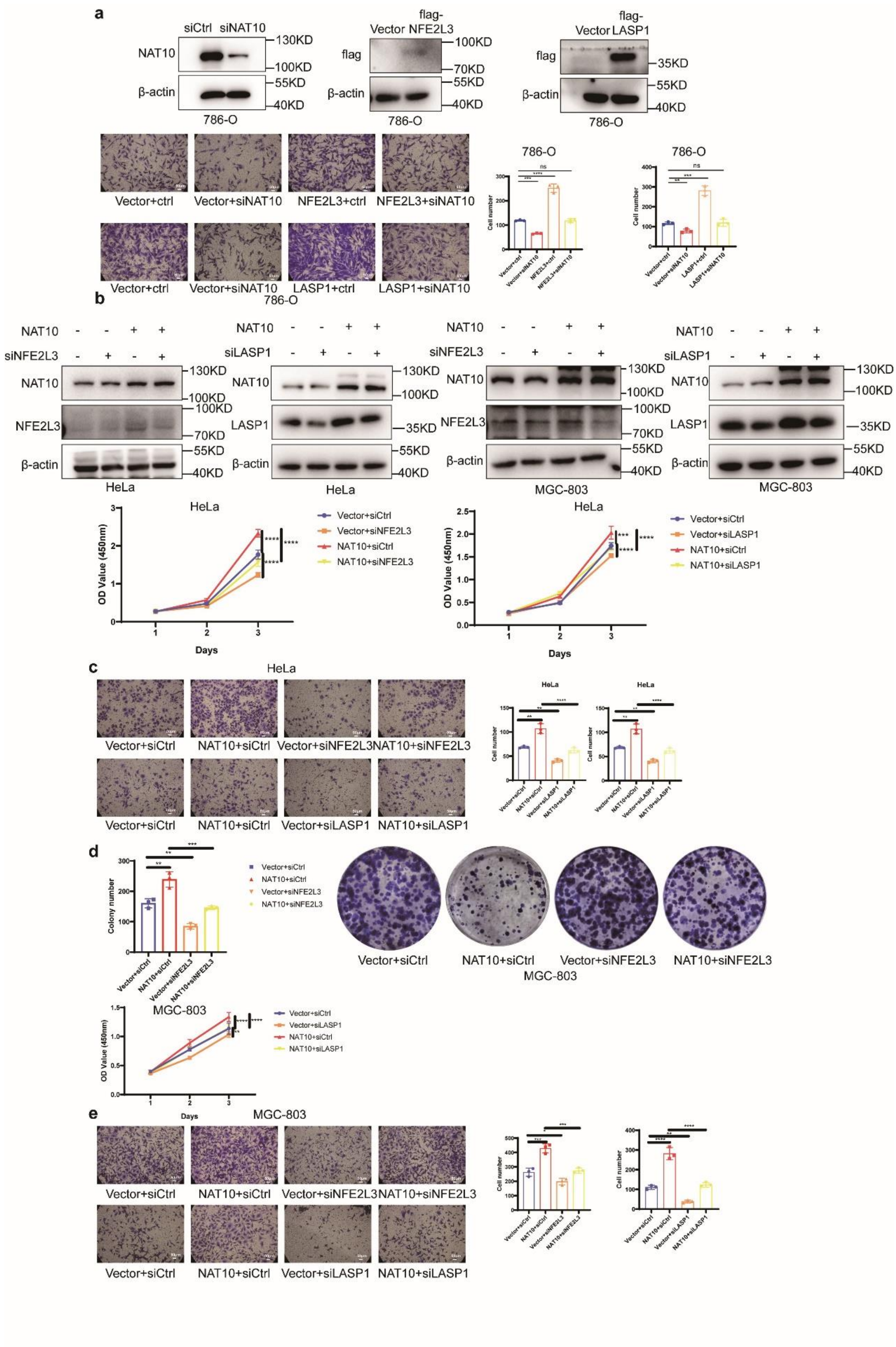

Supplementary figure legends

Supplementary Figure S1

a Flow cytometry showed that the G0/G1 phase of 786-O and A498 cells increased significantly after NAT10 knockdown. **b** Cell scratch assay showed that migration ability was inhibited after NAT10 knockdown in 786-O cells and A498 cells. Scale: 100μm. **c** QRT-PCR was used to detect the overexpression of NAT10 mRNA in A498 cells. **d** Left: NAT10 improved the ability of cell colony generation in A498 cells. Right: quantitative analysis of cell colony generating capacity. **e** Left: EdU results showed that the proliferation ability of A498 cells was significantly improved after NAT10 overexpression. Right: quantitative analysis of EdU^{+} proliferating cells. Scale: 50μm. **f** Left: Transwell assay detected that the migration ability of A498 cells was significantly improved after NAT10 overexpression. Right: quantitative analysis of migrating cells. Scale: 50μm. **g** Expression levels of KIRC NAT10 in the TCGA database on the UALAN website. **h** The expression level of NAT10 in different grades of ccRCC was analyzed by TISID8 database. **i** Left: WT and mut luciferase activity of NAT10 promoter after HIF-1α knockdown; The luciferase activity of NAT10 promoter and mutant promoter changed under hypoxia condition. Right: The effect of knocdown HIF-1α on luciferase activity of NAT10 promoter under normal and hypoxia conditions. **j** Effect of knockdown HIF-1α on NAT10 expression under normal oxygen and hypoxia conditions. **k** In 20 ccRCC tissues, the expression of NAT10 was positively correlated with VEGFA($r=0.8869, p<0.0001$) and LDHA($r=0.8259, p<0.0001$). l IHC results showed that the expression of NAT10 was positively correlated with HIF-1α. m GEPIA database also showed a positive correlation between NAT10 and HIF1α expression in

KIRC. n The inhibition effect of remodelin on acetylation was examined by dot blot, lv-shNAT10 was the positive control.

Supplementary Figure S2

a The heat maps of ac4C modification level in ccRCC group and paracancer tissue group were analyzed. **b** RNA expression of 12 candidate genes after NAT10 knockdown in A498 cells. **c** QRT-PCR showed that NFE2L3 mRNA level decreased after NAT10 knockdown in A498 cells. **d** Dual luciferase reporter assay in A498 cells showed that NAT10 knockdown reduced the luciferase activity of NFE2L3 wild type vector, site 1 mutant and site 2 mutant vector, but had no effect on site 1&2 mutant vector. **e** RNA decay experiment showed that the stability of NFE2L3 mRNA decreased after NAT10 knockdown in A498 cells. **f** The knockdown of NAT10 in A498 cells reduced the translation efficiency of NFE2L3 wild type vector, site 1 and site 2 mutants, but had no effect on site 1&2 mutants. **g** A498 cells were transfected respectively with Flag-NFE2L3-WT, Flag-NFE2L3-mut1 and Flag-NFE2L3-mut2 Flag-NFE2L3-mut1&2 plasmids. Western blot was used to detect the level of Flag in the control group and the NAT10 interference group. **h** Using NAT10 antibody as CO-IP, NAT10 antibody cannot pull down NFE2L3 protein. **i** Colocalization analysis of NAT10 and NFE2L3 mRNA in ccRCC tissues. **j** Colocalization analysis of NAT10 and NFE2L3 mRNA in 786-O. **k** Left: Representative images of in situ NFE2L3 expression levels in 25 pairs of ccRCC cancer tissues and adjacent tissues detected by immunohistochemical staining. Scale: 100μm (left); 50μm (right). Statistical analysis of the intensity of immunohistochemical staining (right). **l** The results of IHC showed that the expression of NFE2L3 was

positively correlated with the expression of NAT10. **m** GEPIA database showed a positive correlation between NAT10 and NFE2L3 expression.

Supplementary Figure S3

a Left: EdU results showed that the proliferation ability of A498 cells was significantly inhibited after NFE2L3 knockdown. Right: quantitative analysis of EdU+ proliferating cells. Scale: 50μm. **b** The proliferation ability of A498 cells was significantly inhibited after NFE2L3 knockdown detected by CCK8. **c** Left: Transwell determined that the migration ability of A498 cells was significantly inhibited after NFE2L3 knockdown. Right: quantitative analysis of migrating cells. Scale: 50μm. **d** Flow cytometry detected that the G0/G1 phase of A498 cells increased significantly after NFE2L3 knockdown, and the histogram showed that the G0/G1 phase cycle arrest occurred after NFE2L3 knockdown.

Supplementary Figure S4

a NFE2L3 and LASP1 promoter binding peak in ChIP-seq (anti-Flag-NFE2L3). **b** The mRNA of NFE2L3 and LASP1 in A498 cells were decreased after NAT10 knockdown by QRT-PCR. **c** QRT-PCR was used to detect the decrease of LASP1 mRNA in A498 cells after NFE2L3 knockdown. **d** Under normal oxygen and hypoxia conditions, the expression of NAT10\NFE2L3\LASP1 axis was detected after HIF-1α knocked down.

Supplementary Figure S5

a Western blot analysis of the effect of knockdown NAT10, overexpressing NFE2L3 and LASP1. Left: Transwell detected 786-O knockdown of NAT10 and migration ability of overexpressed NFE2L3 or LASP1 after knockdown of NAT10. Right: quantitative analysis of migrating cells. Scale: 50μm. **b** Western blot analysis of the effect of knockdown NFE2L3, LASP1 and overexpression of NAT10 in HeLa cells and

gastric cancer cells MGC-803.CCK8 suggests that knocking down NFE2L3 or LASP1 can restore the changes in cell proliferation caused by overexpression of NAT10. **c** Left: Transwell suggests that knocking down NFE2L3 or LASP1 can restore the changes in cell migration caused by overexpression of NAT10. Right: quantitative analysis of migrating cells. Scale: 50μm.**d** Colony formation and CCK8 suggested that knocking out NFE2L3 or LASP1 could restore the proliferation of MGC-803 cells induced by NAT10 overexpression. **e** Left:Transwell suggests that knocking down NFE2L3 or LASP1 can restore the changes in cell migration of MGC-803 cells caused by overexpression of NAT10. Right: quantitative analysis of migrating cells. Scale: 50μm.

Supplementary Table 1

Clinical factors	expression of NAT10(IHC score)		P-value
	Low（n=12）	high（n=31）	
Age（Years）			
≤60	5	21	0.723
> 60	7	10	
Gender			
Female	3	8	1.000
Male	9	23	
Distant metastasis			
M0	7	21	0.723
M1	5	10	
Lymph node			

metastasis			
N0	11	30	0.485
N1+N2+N3	1	1	
T classification			
T1+T2	6	21	0.313
T3+T4	6	10	
WHO/ISUP grade			
I-II	9	11	**0.039**
III-IV	3	20	

Supplementary Table 2

The sequences of siRNAs and primers used in this study.

gene	sense	antisense
siNAT10-1	AUGGAACACUGAACAUAAATT	UUUAUGUUCAGUGUUCCAUTT
siNAT10-2	GGCCAAAGCUGUCUUGAAATT	UUUCAAGACAGCUUUGGCCTT
siHIF-1A-1	GAUGAAAGAAUUACCGAAUTT	AUUCGGUAAUUCUUUCAUCTT
siHIF-1A-2	CGAGGAAGAACUAUGAACATT	UGUUCAUAGUUCUUCCUCGTT
siNFE2L3-1	GGCCUUUCUUUAGAUUCAATT	UUGAAUCUAAAGAAAGGCCTT
siNFE2L3-2	GGGACAGAUACUUCUUUCUTT	AGAAAGAAGUAUCUGUCCCTT
NC siRNA	UUCUCCGAACGUGUCACGUTT	ACGUGACACGUUCGGAGAATT
**RT-qPCR**		
GAPDH	GGCCTCCAAGGAGTAAGACC	AGGGGAGATTCAGTGTGGTG
NAT10	GGGATTGGCCTGCAGCATA	GGCTCCATGACCACATCCTT
HIF-1A	GTGGTAGTGGTGGCATTAGCAGTAG	CCATTAGAAAGCAGTTCCGCAAGC
NFE2L3	GATGTGCGAGCGAGGAGAAT	GGGCTGACACCCTTTCTTCA

CKS1B	TGATCCATGAACCAGAACCTCAC	GTGGTGGTGGCTCTACTCAA
TRIM7	CCATCCTGCTCCAACTCTCC	CGCTGCCATCTGTTTCAGC
DNMT1	CTGCTGAAGCCTCCGAGATG	CAGGTAGCCCTCCTCGGATA
CDHR1	CCGAGCCGTGTCATCCTCT	TGGCCTGAGCCAAGCAG
WNT5B	GCCCCCAATGTAGCCTAGTT	AGAGTAGGGTTCCCTCTGTCA
NOTCH1	GCAAGAACGCCGGGACA	TGCTGGCACGATTTCCCTGA
LAMA3	AGTTCACAGCAGCAAAGGGT	CCAGTGGCAAAGCTGTTAGT
TSPAN4	AGCAACAAGTGGGAGCTTGG	AAGAGCAGGTTGAAGGCGAA
CTSZ	GAACCAGCACATCCCCCAATA	GATGTTGATCCGATCCGCCATA
AHNAK2	CAGCGACTGGACGCGA	AGGCCCTTCAGTCACAGAGT
**acRIP-QPCR**		
NFE2L3-WT	CGAGGGAACGCCTTTGTGC	CCTGCCTGGAACCCAGATAAG
NFE2L3-Mut1	GATGAAGCACCTGAAGCGGTG	ACAGGGTGAACAACAACAGCAG
NFE2L3-Mut2	GCATGGCTGGTGCATAGTG	GGTGGACGAGGCAACAACAAC
NFE2L3-Mut1#2	GCATGGCTGGTGCATAGTGT	GAGGCAACAACAACGCCGA
**RIP-QPCR**		
NFE2L3	GATGTGCGAGCGAGGAGAAT	GGGCTGACACCCTTTCTTCA
**ChIP-QPCR**		
NAT10-promoter	CGCTTCTGGTGGCTTACGTC	GGTAGACAGCACGTGCGCA

Supplementary Table 3

The sequences of Double luciferase experiment

**gene**	
NFE2L3-pmirGLO-WT	GTGCCGGCTGCCGAGGGAACGCCTTTGTGCCCGGTGCTGGGAACCCGCGACGGCCGCCACGCGCCCCGGTCCATTGTTTCGCTTATCTGGGTTCCAGGCAGGTGCGGGCGGCGCGCGGGGTCCGCACGTGTCACCCCGGCGGCTGGGGCGCCGGGACCCGCGGGCGCCGGCAGGGGCGTTCCCGGGCGCGCGGCGGCGATGAAGCACCTGAAGCGGTGGTGGTCGGCCGGCGGCGGCCTCCTGCACCTCACCCTCCTGCTGAGCTTGGCGGGGCTCCGCGTAGACCTAGATCTTTACCTGCTGCTGCCGCCGCCCACC

	CTGCTGCAGGACGAGCTGCTGTTCCTGGGCGGCCCGGCCAGCTC<br>CGCCTACGCGCTCAGCCCCTTCTCGGCCTCGGGAGGGTGGGGGC<br>GCGCGGGCCACTTGCACCCCAAGGGCCGGGAGCTGGACCCTGC<br>CGCGCCGCCCGAGGGCCAGCTGCTCCGGGAGGTGCGCGCGCTC<br>GGGGTCCCCTTCGTCCCTCGCACCAGCGTGGATGCATGGCTGGT<br>GCACAGCGTGGCTGCCGGGAGCGCGGACGAGGCCCACGGGCTG<br>CTCGGCGCCGCCGCCGCCTCGTCCACCGGAGGAGCCGGCGCCA<br>GCGTGGACGGCGGCAGCCAGGCTGTGCAGGGGGGCGGCGGGG<br>ACCCCCGAGCGGCTCGGAGTGGCCCCTTGGACGCCGGGGAAGA<br>GGAGAAGGCACCCGCGGAACCGACGGCTCAGGTGCCGGACGCT<br>GGCGGATGTGCGAGCGAG
NFE2<br>L3-<br>pmirG<br>LO-<br>mut1	GTGCCGGCTGCCGAGGGAACGCCTTTGTGCCCGGTGCTGGGAA<br>CCCGCGA<br>CGGCCGCCACGCGCCCCGGTCCATTGTTTCGCTTATCTGGGTTCC<br>AGGCA<br>GGTGCGGGCGGCGCGCGGGGTCCGCACGTGTCACCCCGGCGGC<br>TGGGGCGCCGGGACCCGCGGGCGCCGGCAGGGGCGTTCCCGGG<br>CGCGCGGCGGCGATGAAGCACCTGAAGCGGTGGTGGTCGGCCG<br>GCGGCGGAAGAAGAAGAACAGCCCTCCTGCTGAGCTTGGCGGG<br>GCTCCGCGTAGACCTAGATCTTTACCTGCTGCTGAAATAAAACAC<br>CCTGCTGCAGGACGAGCTGCTGTTCCTGGGCGGCCCGGCCAGCT<br>CCGCCTACGCGCTCAGCCCC
NFE2<br>L3-<br>pmirG<br>LO-<br>mut2	GTGCGCGCGCTCGGGGTCCCCTTCGTCCCTCGCACCAGCGTGGA<br>TGCATGG<br>CTGGTGCACAGCGTGGCTGCCGGGAGCGCGGACGAGGCCCACG<br>GGCTGCTCGGCGAAATAAATAAATCGTCCACCGGAGGAGCCGGC<br>GCCAGCGTGGACGGCGGCAGCCAGGCTGTGCAGGGGGGCGGCG<br>GGGACCCCCGAGCGGCTCGGAGTGGCCCCTTGGACGCCGGGGA<br>AGAGGAGAAGGCACCCGCGGAACCGACGGCTCAGGTGCCGGAC<br>GCTGGCGGATGTGCGAGCGAG
NFE2<br>L3-<br>pmirG<br>LO-<br>mut1<br>&2	GTGCCGGCTGCCGAGGGAACGCCTTTGTGCCCGGTGCTGGGAA<br>CCCGCGA<br>CGGCCGCCACGCGCCCCGGTCCATTGTTTCGCTTATCTGGGTTCC<br>AGGCA<br>GGTGCGGGCGGCGCGCGGGGTCCGCACGTGTCACCCCGGCGGC<br>TGGGGCGCCGGGACCCGCGGGCGCCGGCAGGGGCGTTCCCGGG<br>CGCGCGGCGGCGATGAAGCACCTGAAGCGGTGGTGGTCGGCCG<br>GCGGCGGAAGAAGAAGAACAGCCCTCCTGCTGAGCTTGGCGGG<br>GCTCCGCGTAGACCTAGATCTTTACCTGCTGCTGAAATAAAACAC<br>CCTGCTGCAGGACGAGCTGCTGTTCCTGGGCGGCCCGGCCAGCT<br>CCGCCTACGCGCTCAGCCCCTTCTCGGCCTCGGGAGGGTGGGGG<br>CGCGCGGGCCACTTGCACCCCAAGGGCCGGGAGCTGGACCCTG<br>CCGCGCCGCCCGAGGGCCAGCTGCTCCGGGAGGTGCGCGCGCT<br>CGGGGTCCCCTTCGTCCCTCGCACCAGCGTGGATGCATGGCTGG

	TGCACAGCGTGGCTGCCGGGAGCGCGGACGAGGCCCACGGGCT<br>GCTCGGCGCCGCCGCCGCCTCGTCCACCGGAGGAGCCGGCGCC<br>AGCGTGGACGGCGGCAGCCAGGCTGTGCAGGGGGGCGGCGGG<br>GACCCCCGAGCGGCTCGGAGTGGCCCCTTGGACGCCGGGGAAG<br>AGGAGAAGGCACCCGCGGAACCGACGGCTCAGGTGCCGGACGC<br>TGGCGGATGTGCGAGCGAG

Supplementary Table 1

Supplementary Table 1

Clinical factors	expression of NAT10(IHC score)		P-value
	Low（n=12）	high（n=31）	
Age（Years）			
≤60	5	21	0.723
＞60	7	10	
Gender			
Female	3	8	1.000
Male	9	23	
Distant metastasis			
M0	7	21	0.723
M1	5	10	
Lymph node metastasis			
N0	11	30	0.485
N1+N2+N3	1	1	
T classification			
T1+T2	6	21	0.313

T3+T4	6	10	
WHO/ISUP grade			
I-II	9	11	**0.039**
III-IV	3	20	

Supplementary Table 2

The sequences of siRNAs and primers used in this study.

**gene**	**sense**	**antisense**
siNAT10-1	AUGGAACACUGAACAUAAATT	UUUAUGUUCAGUGUUCCAUTT
siNAT10-2	GGCCAAAGCUGUCUUGAAATT	UUUCAAGACAGCUUUGGCCTT
siHIF-1A-1	GAUGAAAGAAUUACCGAAUTT	AUUCGGUAAUUCUUUCAUCTT
siHIF-1A-2	CGAGGAAGAACUAUGAACATT	UGUUCAUAGUUCUUCCUCGTT
siNFE2L3-1	GGCCUUUCUUUAGAUUCAATT	UUGAAUCUAAAGAAAGGCCTT
siNFE2L3-2	GGGACAGAUACUUCUUUCUTT	AGAAAGAAGUAUCUGUCCCTT
NC siRNA	UUCUCCGAACGUGUCACGUTT	ACGUGACACGUUCGGAGAATT
**RT-qPCR**		
GAPDH	GGCCTCCAAGGAGTAAGACC	AGGGGAGATTCAGTGTGGTG
NAT10	GGGATTGGCCTGCAGCATA	GGCTCCATGACCACATCCTT
HIF-1A	GTGGTAGTGGTGGCATTAGCAGTAG	CCATTAGAAAGCAGTTCCGCAAGC
NFE2L3	GATGTGCGAGCGAGGAGAAT	GGGCTGACACCCTTTCTTCA
CKS1B	TGATCCATGAACCAGAACCTCAC	GTGGTGGTGGCTCTACTCAA
TRIM7	CCATCCTGCTCCAACTCTCC	CGCTGCCATCTGTTTCAGC
DNMT1	CTGCTGAAGCCTCCGAGATG	CAGGTAGCCCTCCTCGGATA
CDHR1	CCGAGCCGTGTCATCCTCT	TGGCCTGAGCCAAGCAG
WNT5B	GCCCCCAATGTAGCCTAGTT	AGAGTAGGGTTCCCTCTGTCA

NOTCH1	GCAAGAACGCCGGGACA	TGCTGGCACGATTTCCCTGA
LAMA3	AGTTCACAGCAGCAAAGGGT	CCAGTGGCAAAGCTGTTAGT
TSPAN4	AGCAACAAGTGGGAGCTTGG	AAGAGCAGGTTGAAGGCGAA
CTSZ	GAACCAGCACATCCCCCAATA	GATGTTGATCCGATCCGCCATA
AHNAK2	CAGCGACTGGACGCGA	AGGCCCTTCAGTCACAGAGT
**acRIP-QPCR**		
NFE2L3-WT	CGAGGGAACGCCTTTGTGC	CCTGCCTGGAACCCAGATAAG
NFE2L3-Mut1	GATGAAGCACCTGAAGCGGTG	ACAGGGTGAACAACAACAGCAG
NFE2L3-Mut2	GCATGGCTGGTGCATAGTG	GGTGGACGAGGCAACAACAAC
NFE2L3-Mut1#2	GCATGGCTGGTGCATAGTGT	GAGGCAACAACAACGCCGA
**RIP-QPCR**		
NFE2L3	GATGTGCGAGCGAGGAGAAT	GGGCTGACACCCTTTCTTCA
**ChIP-QPCR**		
NAT10-promoter	CGCTTCTGGTGGCTTACGTC	GGTAGACAGCACGTGCGCA

Supplementary Table 3

The sequences of Double luciferase experiment

**gene**	
NFE2L3-pmirGLO-WT	GTGCCGGCTGCCGAGGGAACGCCTTTGTGCCCGGTGCTGGGAACCCGCGACGGCCGCCACGCGCCCCGGTCCATTGTTTCGCTTATCTGGGTTCCAGGCAGGTGCGGGCGGCGCGCGGGGTCCGCACGTGTCACCCCGGCGGCTGGGGCGCCGGGACCCGCGGGCGCCGGCAGGGGCGTTCCCGGGCGCGCGGCGGCGATGAAGCACCTGAAGCGGTGGTGGTCGGCCGGCGGCGGCCTCCTGCACCTCACCCTCCTGCTGAGCTTGGCGGGGCTCCGCGTAGACCTAGATCTTTACCTGCTGCTGCCGCCGCCCACCCTGCTGCAGGACGAGCTGCTGTTCCTGGGCGGCCCGGCCAGCTCCGCCTACGCGCTCAGCCCCTTCTCGGCCTCGGGAGGGTGGGGGCGCGCGGGCCACTTGCACCCCAAGGGCCGGGAGCTGGACCCTGCCGCGCCGCCCGAGGGCCAGCTGCTCCGGGAGGTGCGCGCGCTCGGGGTCCCCTTCGTCCCTCGCACCAGCGTGGATGCATGGCTGGTGCACAGCGTGGCTGCCGGGAGCGCGGACGAGGCCCACGGGCTGCTCGGCGCCGCCGCCGCCTCGTCCACCGGAGGAGCCGGCGCCAGCGTGGACGGCGGCAGCCAGGCTGTGCAGGGGGGCGGCGGGG

	ACCCCCGAGCGGCTCGGAGTGGCCCCTTGGACGCCGGGGAAGA<br>GGAGAAGGCACCCGCGGAACCGACGGCTCAGGTGCCGGACGCT<br>GGCGGATGTGCGAGCGAG
NFE2L3-pmirGLO-mut1	GTGCCGGCTGCCGAGGGAACGCCTTTGTGCCCGGTGCTGGGAA<br>CCCGCGA<br>CGGCCGCCACGCGCCCCGGTCCATTGTTTCGCTTATCTGGGTTCC<br>AGGCA<br>GGTGCGGGCGGCGCGCGGGGTCCGCACGTGTCACCCCGGCGGC<br>TGGGGCGCCGGGACCCGCGGGCGCCGGCAGGGGCGTTCCCGGG<br>CGCGCGGCGGCGATGAAGCACCTGAAGCGGTGGTGGTCGGCCG<br>GCGGCGGAAGAAGAAGAACAGCCCTCCTGCTGAGCTTGGCGGG<br>GCTCCGCGTAGACCTAGATCTTTACCTGCTGCTGAAATAAAACAC<br>CCTGCTGCAGGACGAGCTGCTGTTCCTGGGCGGCCCGGCCAGCT<br>CCGCCTACGCGCTCAGCCCC
NFE2L3-pmirGLO-mut2	GTGCGCGCGCTCGGGGTCCCCTTCGTCCCTCGCACCAGCGTGGA<br>TGCATGG<br>CTGGTGCACAGCGTGGCTGCCGGGAGCGCGGACGAGGCCCACG<br>GGCTGCTCGGCGAAATAAATAAATCGTCCACCGGAGGAGCCGGC<br>GCCAGCGTGGACGGCGGCAGCCAGGCTGTGCAGGGGGGCGGCG<br>GGGACCCCCGAGCGGCTCGGAGTGGCCCCTTGGACGCCGGGGA<br>AGAGGAGAAGGCACCCGCGGAACCGACGGCTCAGGTGCCGGAC<br>GCTGGCGGATGTGCGAGCGAG
NFE2L3-pmirGLO-mut1&2	GTGCCGGCTGCCGAGGGAACGCCTTTGTGCCCGGTGCTGGGAA<br>CCCGCGA<br>CGGCCGCCACGCGCCCCGGTCCATTGTTTCGCTTATCTGGGTTCC<br>AGGCA<br>GGTGCGGGCGGCGCGCGGGGTCCGCACGTGTCACCCCGGCGGC<br>TGGGGCGCCGGGACCCGCGGGCGCCGGCAGGGGCGTTCCCGGG<br>CGCGCGGCGGCGATGAAGCACCTGAAGCGGTGGTGGTCGGCCG<br>GCGGCGGAAGAAGAAGAACAGCCCTCCTGCTGAGCTTGGCGGG<br>GCTCCGCGTAGACCTAGATCTTTACCTGCTGCTGAAATAAAACAC<br>CCTGCTGCAGGACGAGCTGCTGTTCCTGGGCGGCCCGGCCAGCT<br>CCGCCTACGCGCTCAGCCCCTTCTCGGCCTCGGGAGGGTGGGGG<br>CGCGCGGGCCACTTGCACCCCAAGGGCCGGGAGCTGGACCCTG<br>CCGCGCCGCCCGAGGGCCAGCTGCTCCGGGAGGTGCGCGCGCT<br>CGGGGTCCCCTTCGTCCCTCGCACCAGCGTGGATGCATGGCTGG<br>TGCACAGCGTGGCTGCCGGGAGCGCGGACGAGGCCCACGGGCT<br>GCTCGGCGCCGCCGCCGCCTCGTCCACCGGAGGAGCCGGCGCC<br>AGCGTGGACGGCGGCAGCCAGGCTGTGCAGGGGGGCGGCGGG<br>GACCCCCGAGCGGCTCGGAGTGGCCCCTTGGACGCCGGGGAAG<br>AGGAGAAGGCACCCGCGGAACCGACGGCTCAGGTGCCGGACGC<br>TGGCGGATGTGCGAGCGAG

Supplementary material 2

***RNA-Seq* data analysis**

Raw sequencing data was first filtered by Trimmomatic (version 0.36), low-quality reads were discarded and the reads contaminated with adaptor sequences were trimmed. Clean Reads were further treated with in-house scripts to eliminate duplication bias introduced in library preparation and sequencing. In brief, clean reads were first clustered according to the UMI sequences, in which reads with the same UMI sequence were grouped into the same cluster. Reads in the same cluster were compared to each other by pairwise alignment, and then reads with sequence identity over 95% were extracted to a new sub-cluster. After all sub-clusters were generated, multiple sequence alignment was performed to get one consensus sequence for each sub-clusters. After these steps, any errors and biases introduced by PCR amplification or sequencing were eliminated.

The de-duplicated consensus sequences were used for standard RNA-seq analysis. They were mapped to the reference genome of *homo sapiens* from ftp://ftp.ensembl.org/pub/release-87/fasta/homo_sapiens/dna/) using STAR software (version 2.5.3a) with default parameters. Reads mapped to the exon regions of each gene were counted by featureCounts (Subread-1.5.1; Bioconductor) and then RPKM was calculated. Genes differentially expressed between groups were identified using the edgeR package (version 3.12.1). A p-value cutoff of 0.05 and Fold-change cutoff of 2 were used to judge the statistical significance of gene expression differences. Gene ontology (GO) analysis and Kyoto encyclopedia of genes and genomes (KEGG) enrichment analysis for differentially expressed genes were both implemented by KOBAS software (version: 2.1.1) with a P-value cutoff of 0.05 to judge statistically significant enrichment. Alternative splicing events were detected by using rMATS (version 3.2.5) with a FDR value cutoff of 0.05 and an absolute value of $\Delta\psi$ of 0.05.

ChIP-seq Data analysis

Raw sequencing data was first filtered by Trimmomatic (version 0.36), low-quality reads were discarded and the reads contaminated with adaptor sequences were trimmed.

The clean reads were used for protein binding site analysis. They were mapped to the reference genome of *homo sapiens* from ftp://ftp.ensembl.org/pub/release-87/fasta/homo_sapiens/dna/ using STAR software (version 2.5.3a) with default parameters. The RSeQC（version 2.6）was used for reads distribution analysis.The MACS2 software（Version 2.1.1）was used for peak calling. The bedtools（Version 2.25.0）was used for peaks annotation and peak distribution analysis. The differentially binding peaks were identified by a python script，using fisher test. The Homer（version 4.10）was used for motifs analysis. Gene ontology (GO) analysis and Kyoto encyclopedia of genes and genomes (KEGG) enrichment analysis for annotated genes were both implemented by KOBAS software (version: 2.1.1) with a corrected P-value cutoff of 0.05 to judge statistically significant enrichment.

acRIP-seq Data analysis

Briefly, paired-end reads were harvested from Illumina HiSeq 4000 sequencer, and were quality controlled by Q30. After 3' adaptor-trimming and low quality reads removing by cutadapt software (v1.9.3). The reads were aligned to the reference genome (UCSC HG19) with hisat2 software (v2.0.4). MACS software (v1.4.2) was used for ac4C peak calling. DiffReps software was used to identify differentially enriched ac4C peaks. Peaks were annotated with home-made scripts. GO and Pathway enrichment analysis were performed for the differentially ac4C genes. The read alignments on genome could be visualized with the popular IGV software.
